# Hydride Migration within RhH_2_Ag_19_ Superatom: A Combined Neutron Diffraction and DFT Analysis

**DOI:** 10.1002/smll.202501583

**Published:** 2025-03-23

**Authors:** Tzu‐Hao Chiu, Michael N. Pillay, Jian‐Hong Liao, Xiaoping Wang, Hao Liang, Samia Kahlal, Jean‐Yves Saillard, C. W. Liu

**Affiliations:** ^1^ Department of Chemistry National Dong Hwa University No. 1, Sec. 2, Da Hsueh Rd. Hualien 97401 Taiwan; ^2^ Neutron Scattering Division Neutron Sciences Directorate Oak Ridge National Laboratory Oak Ridge Tennessee 37831 USA; ^3^ Univ Rennes, CNRS, ISCR‐UMR 6226 Rennes 35000 France

**Keywords:** hydrides, isomers, migration, rhodium, silver

## Abstract

An investigation combining neutron diffraction and DFT allows determining the most likely hydride migration pathway within the icosahedral metal framework of [RhH_2_Ag_19_{S_2_P(O*^n^* Pr)_2_}_12_] (**RhH_2_Ag_19_**). Starting from the experimentally derived solid‐state structures, a computational analysis is able to reveal an energetically favorable migration pathway with a maximum energy barrier of 4.2 kcal mol^−1^. The two hydrides migrate simultaneously within the Rh@Ag_12_ icosahedral core, traversing several positional isomers. This study expands the understanding of hydride dynamics in nanoclusters and provides critical insights into the structural flexibility of the superatom framework. These findings have significant implications for hydrogen storage, catalysis, and the design of advanced hydride‐containing materials.

## Graphical abstract

By modifying the crystallization conditions, the migration pathway of interstitial hydrides is visualized for the first time and analyzed through neutron diffraction experiments combined with DFT calculations. The computational results reveal an approximate migration energy barrier of 4.2 kcal mol^−1^, providing a reasonable explanation for the experimentally observed isomerization phenomenon.

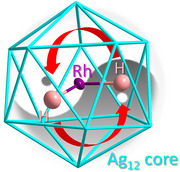

## 1 Introduction

Atomically precise nanoclusters (NCs), unlike traditional nanoparticles with their broader size and structure distribution, offer a significantly more controllable platform for investigating the intricate interplay between structure, properties, and performances. This enables fine‐tuning NC properties, making them highly attractive for diverse applications such as (electro)catalysis, sensing, and biomedicine.^[^ ^1^, ^2^, ^3^ ^]^ As a result, developing novel NCs has become a focal point of research, motivated by the need to tune the NC properties for future advancements.^[^ ^4^ ^]^ Additionally, hydride‐containing NCs offer a platform for physically quantifying metal‐hydride interactions at the atomic level at resolutions not possible in bulk metal‐hydrides.^[^ ^5^ ^]^ Identification of the hydride positions within NCs is crucial, and single‐crystal X‐ray diffraction (SCXRD) has proven valuable in this endeavor.^[^ ^6^ ^]^ Distortions in the metallic framework, such as changes in bond lengths or lattice expansion, can also serve as evidence for assigning hydride location.

Synthetically, doping silver or gold NCs with hetero‐metals like palladium, platinum, or rhodium in the presence of borohydride as a reducing agent in a one‐pot synthesis can induce hydrogen inclusion. Such hydrogen atom(s) lie(s) within the cluster core near the central hetero‐metal and behave(s) somewhat as metallic in nature,^[^ ^7^ ^]^ playing a crucial role in the formation of superatoms.^[^ ^8^ ^]^ Hydride‐containing gold‐rich systems have been initially reported, with gold adept at stabilizing several heterometallic cores.^[^ ^9^, ^10^, ^11^, ^12^, ^13^ ^]^ In terms of silver, comparatively fewer reports have been made to date. Our research group has successfully isolated examples, incorporating PdH, PtH, RhH, and RhH_2_ into M@Ag_19_(dtp/dsep)_12_ clusters (M = PdH, PtH, RhH_2_; dtp = dithiophosphate; dsep = diselenophosphate) and (RhH)@Ag_20_(dtp)_12_, all protected by dichalcophosphate ligands.^[^ ^14^, ^15^, ^16^, ^17^, ^18^, ^19^, ^20^, ^21^ ^]^ Likewise, Lee et al. have reported thiolate‐stabilized NCs incorporating RhH, IrH, RuH_2_, and OsH_2_ into the Ag_12_ icosahedral core of Ag_25_(SR)_18_ clusters.^[^ ^22^, ^23^ ^]^

Importantly, in several cases, we have noted the ability of the hydride in solution, often resulting in complex distribution of isomers. Isomers differing in the positions of their hydrides are common and can sometimes interconvert via hydride migration.^[^ ^24^, ^25^, ^26^, ^27^, ^28^, ^29^ ^]^ Indeed, weak interactions between hydrides and metals often lead to a dynamic hydride migration process. Although this migration was recently suggested from a density functional theory (DFT) investigation on bare (unligated) MHAg_12_ cluster models,^[^ ^30^ ^]^ efforts to experimentally reveal such processes are frequently hindered by the difficulty of capturing intermediate states. Recently, the replacement of hydrides with other more strongly bonded ligands to capture migration intermediates has been reported in Au_22_.^[^ ^31^ ^]^ However, capturing hydride migration intermediates within superatomic cores remains challenging, as such hydrides cannot be replaced by other ligands. We have previously observed from low‐temperature NMR studies that hydride can migrate inside NC icosahedral metallic kernels, leading to complex isomeric distributions in solution.^[^ ^18^ ^]^ The movement is made possible by the unique arrangement of atoms inside the centered icosahedral NC kernel, which offers several tetrahedral interstitial sites that are energetically favorable for hydrogen occupancy. The relatively low energy barriers connecting the occupation of these sites, combined with the inherent flexibility of the icosahedral structure, facilitate hydride migration or “hopping.” Importantly, the occupancy of these interstitial sites leads to measurable distortions in the metallic framework, a key factor we leverage in the following discussion to identify the hydride positions.

We report below a neutron diffraction analysis of a new crystal polymorph of the dihydride NC [RhH_2_Ag_19_{S_2_P(O*^n^* Pr)_2_}_12_] (**RhH_2_Ag_19_**). In combination with SCXRD data and DFT calculations, this study provides insights into the hydride migration process within the icosahedral core. These findings not only expand the library of available hydride‐encapsulating NCs but also contribute to our understanding of hydrogen behavior within metal‐rich systems.

**Scheme 1:**
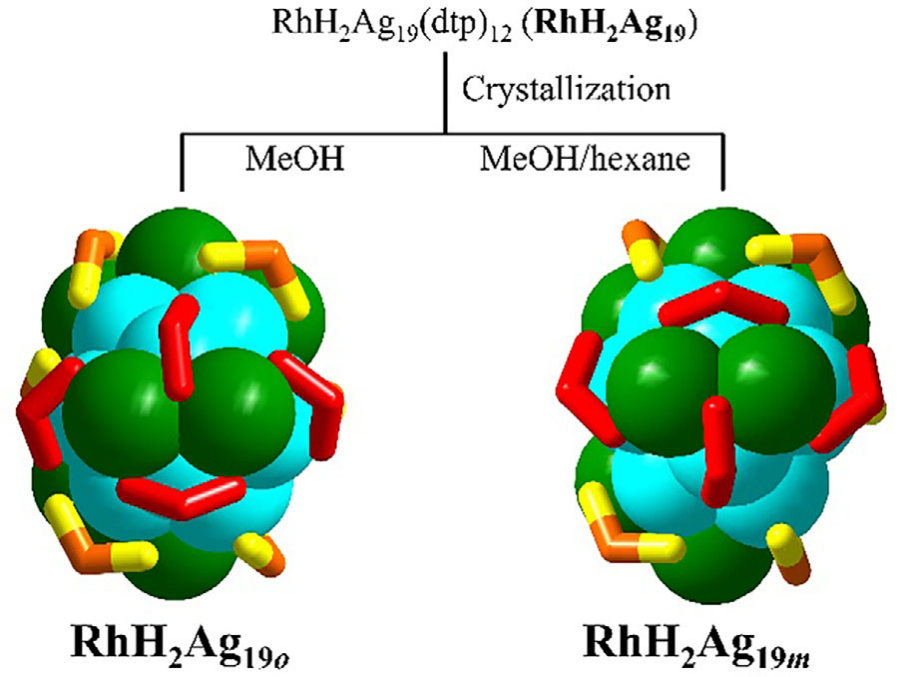
The two *C*_1_ polymorphs of RhH_2_Ag_19_ obtained via different crystallization conditions and characterized by neutron diffraction (RhH_2_Ag19_oN_, orthorhombic phase (this work); RhH_2_Ag19_mN_, monoclinic phase.^[^ ^18^ ^]^ Their configurational differences in ligand layer (S_2_P) is highlighted by the red sticks. Color code: sky blue Ag_ico_, green for Ag_cap_, yellow for S, orange for P.

In the preparation of heterometallic NCs, the solubility of the metal precursors is crucial. As detailed in a previous report,^[^ ^18^ ^]^ **RhH_2_Ag_19_** was synthesized using a co‐reduction method. Organic‐soluble metal precursors, [Ag(MeCN)_4_]BF_4_ and [Rh(COD)Cl]_2_, were reduced by LiBH_4_ in the presence of (NH_4_)[S_2_P(O*^n^* Pr)_2_], (molar ratio: Ag^+^:Rh^+^:[S_2_P(O*^n^* Pr)_2_]¯:H¯ of 40:1:10:20). This reaction yielded [RhH_2_Ag_19_{S_2_P(O*^n^* Pr)_2_}_12_] (**RhH_2_Ag_19_**), which was isolated in pure form by column chromatography. Modifying the crystallization conditions of **RhH_2_Ag_19_** (**Scheme** **1**), we were able to obtain a new crystal habit in the orthorhombic *Pbca* space group (**RhH_2_Ag_19_ *_o_***)^[^ ^32^ ^]^ (Figure S1, Supporting Information), distinct from the previously reported monoclinic *P*2_1/c_ structure (**RhH_2_Ag_19_ *_m_***).^[^ ^18^ ^]^ The molecular structure of **RhH_2_Ag_19_ *_o_*** differs from that of **RhH_2_Ag_19_** ***_m_*** in the orientation and connectivity of four dtp ligands, yielding polymorphs (Scheme 1).

**Figure 1:**
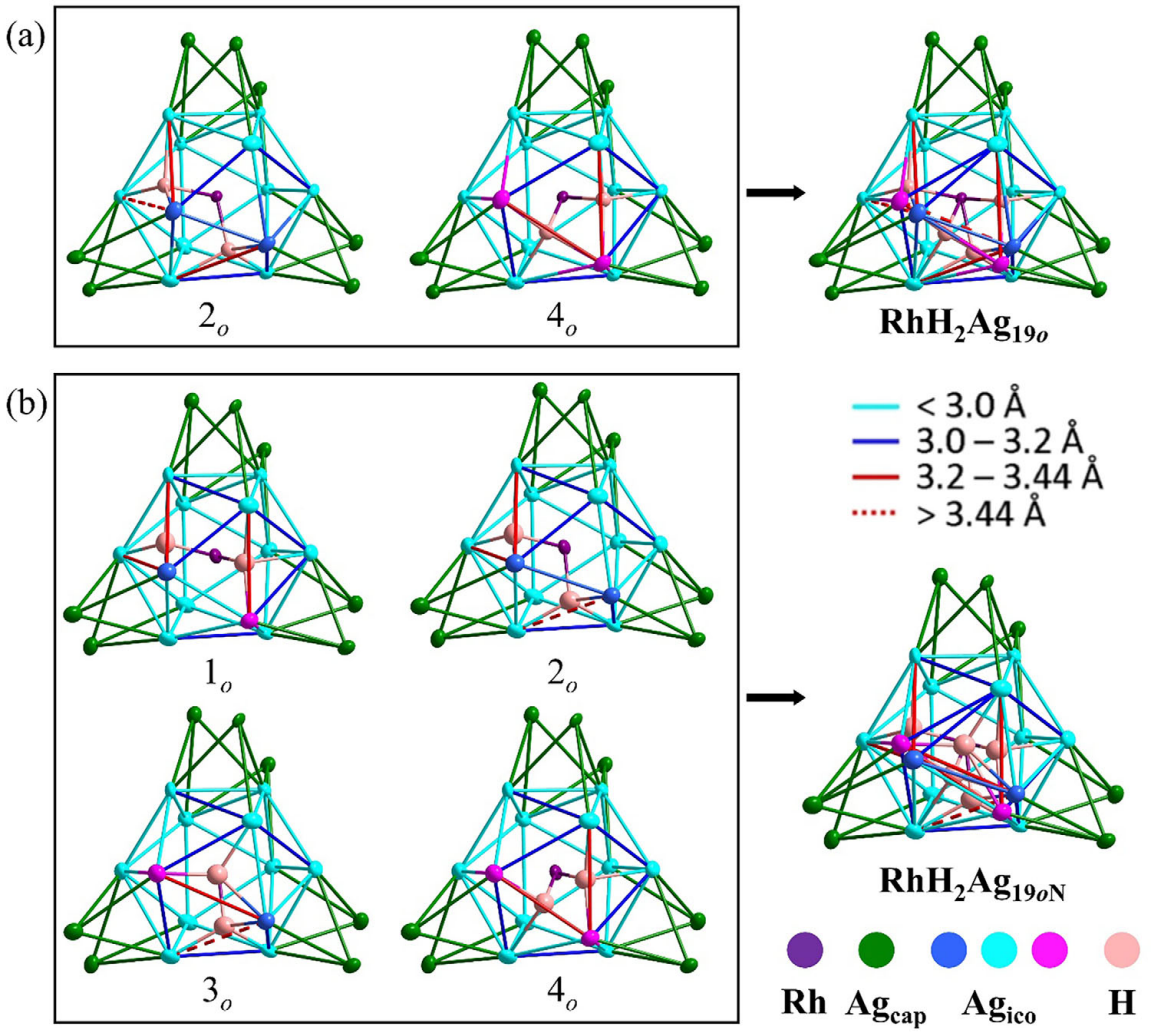
Metal‐framework of a) **RhH_2_Ag_19_ *_o_*** b) **RhH_2_Ag_19_ *_o_*_N_**, with their disorder deconvolution in individual molecular structures.

Recently, we noted the dynamic behavior of the hydrides in solution, characterized by VT‐NMR (Figure S2, Supporting Information)^[^ ^18^ ^]^ Crucially, the disorder observed within the crystal lattices, as captured by neutron (**RhH_2_Ag_19_ *_o_*_N_**) and X‐ray diffraction, provides experimental support for a likely hydride migration pathway within metallic frameworks. In the solid‐state structures, the hydride atoms tend to avoid occupying interstitial sites directly adjacent to capping metal atoms (Ag_cap_). This preference arises from the increased rigidity of the capped triangular faces of the Rh@(Ag_ico_)_12_ centered icosahedral kernel. These specific faces, along with their capping atoms, constitute the green (Ag_ico_)_3_Ag_cap_ tetrahedra shown in Figure S1 (Supporting Information). In contrast, the hydride atoms prefer occupying the most deformable interstitial sites that involve Ag_ico_ atoms distant from Ag_cap_.^[^ ^6^ ^]^ The initial evidence of the hydride positions was attained by first analyzing the distortions of the uncapped (Ag_ico_)_3_ faces in the disordered X‐ray structure of **RhH_2_Ag_19_ *_o_***, which exhibits Ag_ico_ partial occupancies that resolve to 55% and 45% (structures **2*_o_*** and **4*_o_*** in **Figure** **1**a; Figure S3, Supporting Information). Furthermore, the configurations of **2*_o_*** and **4*_o_*** are not identical, and their ∠HRhH angles are different. **2*_o_*** shows an angle (102°) comparable to that observed in **RhH_2_Ag_19_ *_m_***  (101° for both X‐ray and neutron structures), while **4*_o_*** presents a smaller angle (69°). The **RhH_2_Ag_19_ *_o_*_N_** neutron structure is also disordered but differently from its X‐ray **RhH_2_Ag_19_ *_o_*** counterpart. Such a difference can be explained by the different conditions under which the corresponding single crystals were grown. Two disordered Ag_ico_ can result in four slightly different metal frameworks, with the hydride positioned within the expanded tetrahedral cavities of these four distinct metal frameworks. The two hydrides in **RhH_2_Ag_19_ *_o_*_N_** exhibit disorder over five positions, with occupancies of 0.6, 0.43, 0.36, 0.35, and 0.26, respectively, yielding to the four different configurations **1*_o_***, **2*_o_*, 3*_o,_*** and **4*_o_*** shown in Figure 1b, with ∠HRhH angles of 93°, 96°, 66° and 46°, respectively (**Table** **1**). Note that **2*_o_*** and **4*_o_*** are present in both X‐ray and neutron structures. The Rh…H distances range from 1.67(6) to 1.86(10) Å, with an average of 1.73(7) Å, which is comparable to that observed in **RhH_2_Ag_19_ *_m_***.^[^ ^18^ ^]^

**Table 1:** Selected experimental distances (Å) and angles (deg.) for X‐ray and neutron structure **1*_o_***, **2*_o_***, **3*_o_***, and **4*_o_***.

Comp.	1*_o_* [neutron]	2*_o_* [neutron]	3*_o_* [neutron]	4*_o_* [neutron]	2*_o_* [X‐ray]	4*_o_* [X‐ray]
**CSM**	0.37	0.43	0.29	0.22	0.43	0.28
**Rh–Ag_ico_**	2.75(2)‐2.92(4) avg. 2.83(3)	2.75(2)‐2.92(4) avg. 2.83(3)	2.75(2)‐2.91(3) avg. 2.82 (3)	2.76(2)‐2.90(2) avg. 2.82 (3)	2.770(1)‐2.883(2) avg. 2.822(3)	2.770(1)–2.871(2) avg. 2.819(3)
**Ag_ico_–Ag_ico_**	2.82(2)–3.43(3) avg. 2.98(3)	2.79(3)–3.46(3) avg. 2.97(3)	2.79(3)–3.46(3) avg. 2.97(3)	2.82(2)–3.38(3) avg. 2.95(3)	2.799(2)–3.483(2) avg. 2.968(2)	2.819(1)–3.423(2) avg. 2.963(2)
**Ag_ico_–Ag_cap_**	2.89(2)–3.16(3) avg. 3.04(3)	2.89(2)–3.16(3) avg. 3.04(3)	2.89(2)–3.11(2) avg. 3.03(3)	2.89(2)–3.10(2) avg. 3.03(3)	2.886(1)–3.230(1) avg. 3.027(2)	2.886(1)–3.081(1) avg. 3.014(1)
**Rh–H**	1.67(6)–1.72(4) avg. 1.69(5)	1.67(6)–1.72(4) avg. 1.69(5)	1.67(6)–1.71(6) avg. 1.69(6)	1.67(7)–1.86(10) avg. 1.79(8)	1.64 (4)‐1.77(10) avg. 1.71 (10)	1.62(4)–1.63(4) avg. 1.63(6)
**Ag_ico_–H**	1.77(8)–2.27(7) avg. 1.96(7)	1.81(6)–2.09(7) avg. 1.95(6)	1.80(6)–2.09(7) avg. 1.92(6)	1.70(9)–2.26(10) avg. 1.95(9)	1.84 (10)‐2.06(10) avg. 1.94 (18)	1.89(5)–1.94(4) avg. 1.91(10)
**H–Rh–H**	93	96	66	46	102	69

**Figure 2:**
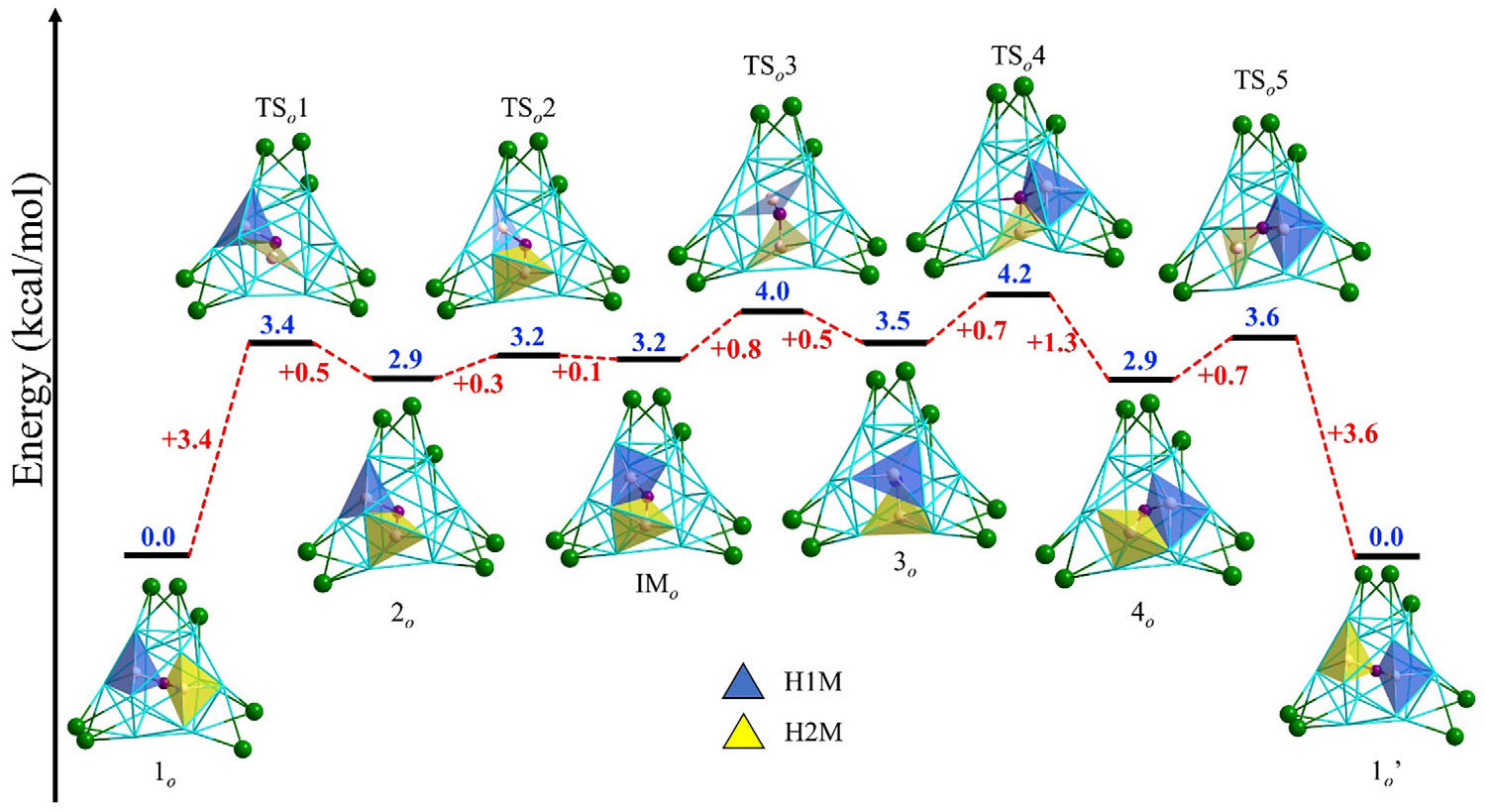
Proposed hydride migration within icosahedron, with configurations **1*_o_***, **2*_o_***, **3*_o_***, and **4*_o_*** observed experimentally (ligands omitted for clarity).

**Figure 3:**
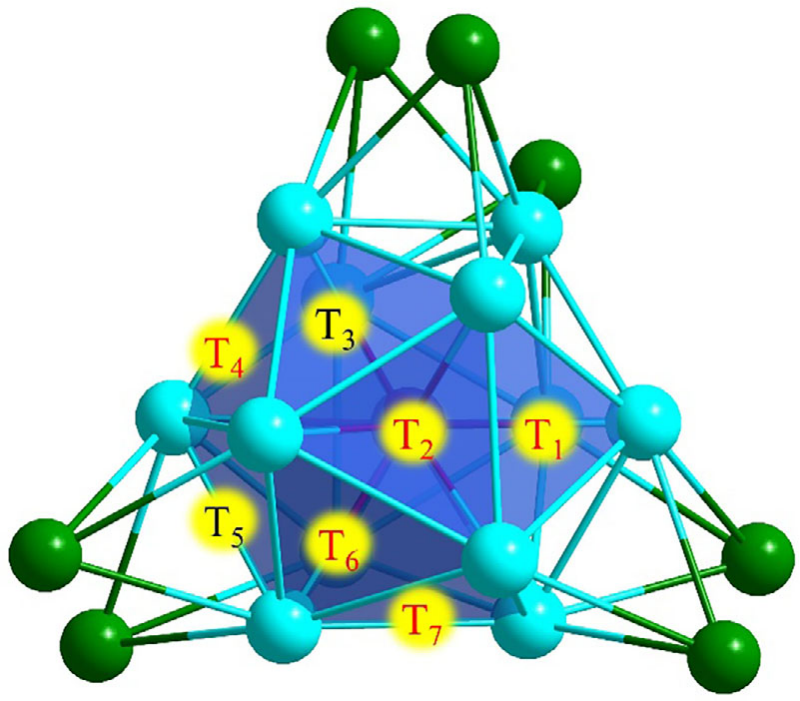
The seven tetrahedral cavities of **RhH_2_Ag_19_ *_o_*** selected for the DFT optimization study with five tetrahedra determined experimentally T1, T2, T4, T6, and T7 (red text); two assigned based on proximity T3, T5 (black text). The tetrahedral cavity where the hydride is located in the solid‐state structure: 1o: (T1, T4), 2o: (T7, T4), 3o: (T2, T7), 4o: (T1, T6).

**Figure 4:**
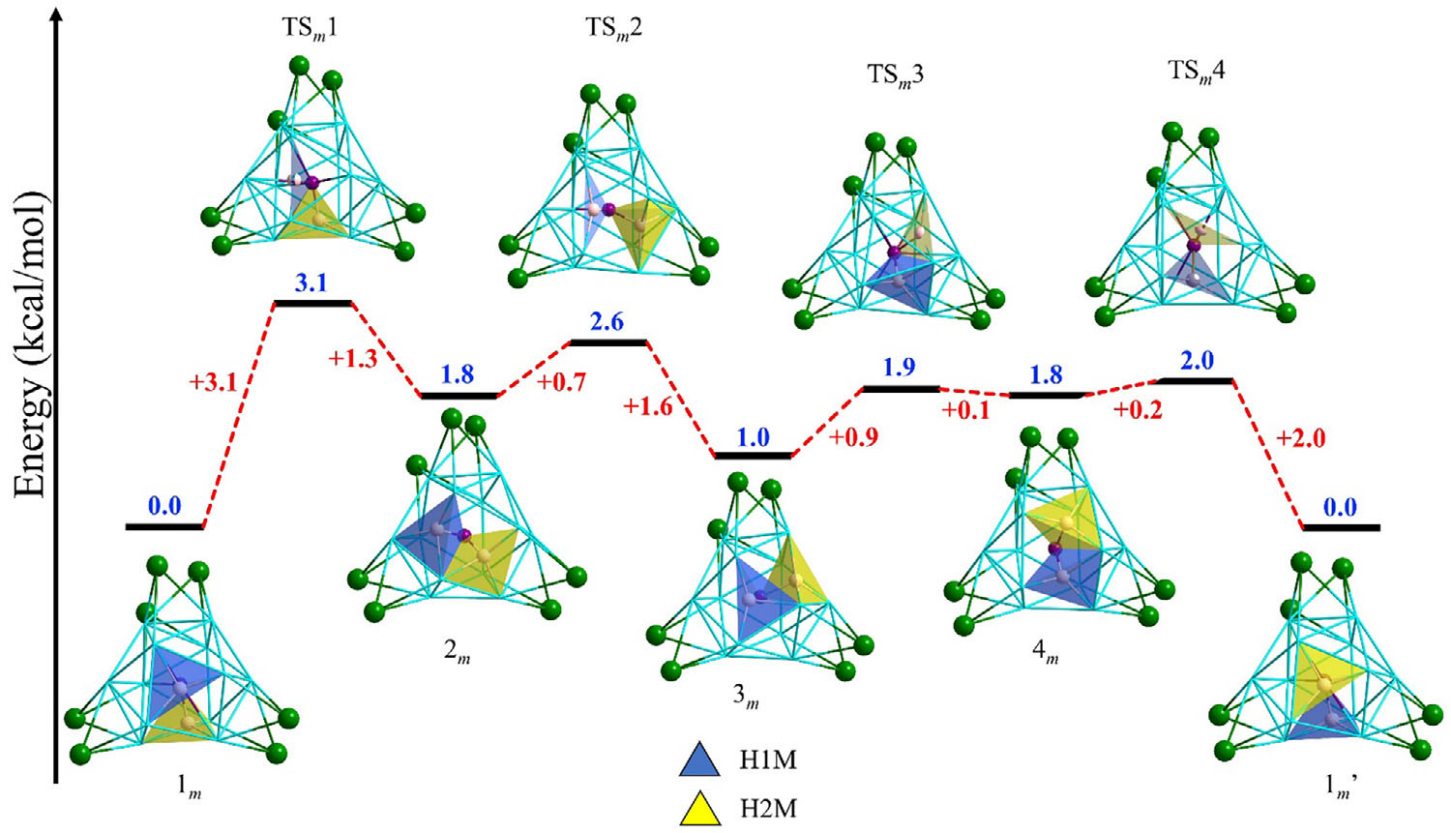
Hydride migration of **RhH_2_Ag_19_ *_m_***. (ligands omitted for clarity).

Starting from their solid‐state structure as models, the geometries of the four distinct configurations **1*_o_***, **2*_o_***, **3*_o_***, and **4*_o_***, were optimized through density functional theory (DFT) calculations. For the sake of computational limitations, the dtp ligands were replaced by S_2_PH_2_ models, a simplification that has been proved to be reasonable in many past investigations.^[^ ^14^, ^15^, ^16^, ^17^, ^18^, ^19^, ^20^ ^]^ The four structures were found to be minima on the potential energy surface and, thus, are *true isomers*. The four isomers lie within a range of only 3.5 kcal mol^−1^, isomer **1*_o_*** being the global minimum (**Figure** **2**). The hydride connectivity in the DFT‐optimized structure of **2*_o_*** slightly differs from that observed in its solid‐state counterpart. In the solid‐state structures of **2*_o_*** (Figure S4, Supporting Information), the two hydrides occupy the T4 and T7 RhAg_3_ tetrahedral cavities identified in **Figure** **3**, whereas, in the optimized structure, they occupy the T4 and T6 cavities, T6 and T7 being neighboring interstitial sites. The discrepancy between the optimized and experimental structures can be attributed to the flatness of the potential energy surface associated with hydride displacements, rendering the ∠HRhH angle sensitive to any weak structural perturbation. To address this, optimizations of the only hydride positions within a frozen cluster cage having the experimental metrics were performed. Indeed, they show a better alignment of the computed results with the experimental ones (Tables S1 and S2, Supporting Information). In particular, the hydrides of the **2*_o_*** (partly) optimized structure now occupy the T4 and T7 cavities. A plausible hydride migration pathway was subsequently explored by investigating interconversions between these four closely related isomers. During our search for transition states, a fifth isomer, namely **IM*_o_***, was discovered. Although this key intermediate has not been experimentally observed, it plays a crucial role in linking the entire migration pathway (Figure 2). The whole hydride migration pathway involves seven different tetrahedral cavities (Figure 3). In each transition state, the imaginary frequency corresponds to the vibration of the μ_3_‐hydride, oscillating between two tetrahedral cavities within the metal framework (Figure S5, Supporting Information). The energy barriers connecting the isomers range as low as nearly 0 to 3.6 kcal mol^−1^ (Figure 2). These small energy differences help explain the dynamic disorder observed experimentally. Notably, as the two hydrides migrate from **1*_o_*** to **2*_o_***, **IM*_o_***, **3*_o_***, and **4*_o_***, and eventually return to **1*_o_*’**, their positions are exchanged. This observation indicates that the two hydrides are equivalent (Figure S6, Supporting Information). Based on the hydride migration pathway of **RhH_2_Ag_19_ *_o_***, we thus propose a possible hydride migration pathway for **RhH_2_Ag_19_ *_m_***. In addition to **1*_m_*** , which shows good alignment with both neutron and X‐ray structures (**RhH_2_Ag_19_ *_m_*_N_** and **RhH_2_Ag_19_ *_m_*** ), we identified three additional distinct intermediates: **2*_m_*** , **3*_m_*** , and **4*_m_*** (**Figure** **4**; Table S3, Supporting Information). Similar results were observed in the hydride migration pathway of **RhH_2_Ag_19_ *_m_*** , characterized by minimal energy barriers and the ability of two hydrides to exchange positions via migration (Figure 4; Figure S7, Supporting Information).

The TD‐DFT‐simulated UV–vis spectra computed at the CAM‐B3LYP/Def2‐TZVP level for all the isomers of **RhH_2_Ag_19_ *_o_*** and **RhH_2_Ag_19_ *_m_***  are shown in Figures S8 and S9 (Supporting Information). The simulated spectra for each isomer have two bands, the one at the lowest energy being sometimes reduced to a shoulder peak. These two bands are of 1P → 1D nature. The variations in the peak profile for each isomer highlight the significant influence of minor geometrical differences on the optical/absorption properties. Notably, when the simulated spectra of all the isomers are averaged, the resulting spectrum shows a good correlation to the experimental spectrum of **RhH_2_Ag_19_** (Figure S9, Supporting Information). This suggests that **RhH_2_Ag_19_** contains a mixture of isomers in solution, correlating to the proposed behavior described herein.

In summary, we obtained isomers of RhH_2_Ag_19_[S_2_P(OPr)_2_]_12_ by controlling crystallization conditions and determined its atomically precise structure through X‐ray and neutron diffraction. For the first time, a visual slice of hydride migration pathways within an enclosed icosahedral core of atomically precise nanoclusters is reported. The experimentally observed lattice disorder supports a possible mechanism for the hydride migration predicted by DFT calculations. This work thus provides valuable justification for future studies that may include dynamic techniques, such as variable‐temperature neutron diffraction or quasielastic neutron scattering, potentially providing direct confirmation of hydride motion. This contributes to our understanding of hydride‐metal interactions and the design of advanced hydride‐containing superatomic systems for tailored applications.

## 2 Experimental Section

All chemicals were purchased from commercial sources and used as received. Solvents were purified following standard protocols. All reactions were carried out under N_2_ atmosphere by using standard Schlenk techniques. [RhH_2_@Ag_19_{S_2_P(O*^n^* Pr)_2_}_12_] was prepared by the same procedure reported earlier.^[^ ^18^ ^]^ The purified product was recrystallized under two different conditions: pure MeOH and a MeOH:hexane (1:1) mixture. Under the pure MeOH condition, black rod‐like crystals of **RhH_2_Ag_19_ *_o_*** were obtained after approximately one week. Under the MeOH:hexane (1:1) condition, black prismatic crystals of **RhH_2_Ag_19_ *_m_***  were obtained after approximately one week.

### X‐Ray Crystallography

Single crystals suitable for X‐ray diffraction analysis of **RhH_2_Ag_19_ *_o_*** was obtained by evaporating MeOH solution at 4 °C within a week. The single crystals were mounted on the tip of glass fiber coated in paratone oil, then frozen. Data were collected on a Bruker APEX II CCD diffractometer using graphite monochromated Mo *K*α radiation (λ = 0.71073 Å) at 100 K. Absorption corrections for area detector were performed with SADABS^[^ ^32^ ^]^ and the integration of raw data frame was performed with SAINT.^[^ ^33^ ^]^ The structure was solved by direct methods and refined by least‐squares against *F* ^2^ using the SHELXL‐2018/3 package,^[^ ^34^, ^35^ ^]^ incorporated in SHELXTL/PC V6.14.^[^ ^36^ ^]^ All non‐hydrogen atoms were refined anisotropically. Selected X‐ray crystallographic data is listed in Table S5 (Supporting Information).

### Single‐Crystal Neutron Diffraction

The hydride locations in [RhH_2_Ag_19_{S_2_P(O*^n^* Pr)_2_}_12_] (**RhH_2_Ag_19_ *_o_*_N_**) were confirmed by a single‐crystal neutron diffraction experiment using the TOPAZ single‐crystal neutron time‐of‐flight (TOF) Laue diffractometer at ORNL's Spallation Neutron Source.^[^ ^37^ ^]^ Single crystals suitable for neutron diffraction were obtained by slow evaporation of methanol at ambient temperature within two weeks. A black, block‐shaped crystal of **RhH_2_Ag_19_ *_o_*_N_** (2.95 mm × 1.06 mm × 0.85 mm, Figure S10, Supporting Information) was attached to a MiTeGen loop using a perfluorinated grease (Krytox GPL 205) and transferred to TOPAZ goniometer for data collection at 100 K. A total of 28 crystal orientations optimized with CrystalPlan software^[^ ^38^ ^]^ were used to ensure better than 95% coverage of a hemisphere of reciprocal space (Figure S12, Supporting Information). The data were collected for ≈136 h with 20 C of proton charge each at the beam power of 1.4 MW. Raw peaks intensities were obtained using the 3‐D ellipsoidal Q‐space integration method available in Mantid.^[^ ^39^ ^]^ Data normalization, including Lorentz, neutron 6 TOF spectrum, and detector efficiency corrections, were carried out with the ANVRED3 program.^[^ ^40^ ^]^ A Gaussian numerical absorption correction was applied with μ = 1.1716 + 1.1088 λ cm^−1^. The reduced data were saved in SHELX HKLF2 format, in which the neutron wavelength for each reflection was recorded separately. Non‐hydrogen atom positions in the X‐ray structure were used for the initial refinement of the neutron structure. Hydrogen atoms on carbon atoms are placed using the riding model available in SHELXL‐2014.^[^ ^41^ ^]^ The position of hydride was located from the difference Fourier map calculated using the neutron data. All non‐hydrogen atoms and the interstitial hydride atoms were refined anisotropically, and the neutron structure was then refined successfully to convergence using the SHELXL‐2014 program. Selected neutron crystallographic data is listed in Table S6 (Supporting Information). The neutron structures reported herein have been deposited at the Cambridge Crystallographic Data Centre. CCDC 2410768 (**RhH_2_Ag_19_ *_o_*_N_**) contains the supplementary crystallographic data. This data can be obtained free of charge from the Cambridge Crystallographic Data Centre via https://www.ccdc.cam.ac.uk/data_request/cif.

### Computational Details

Geometry optimizations were carried out within the formalism of the density functional theory (DFT) with the Gaussian 16 package,^[^ ^42^ ^]^ using the BP86 functional^[^ ^43^, ^44^ ^]^ and the Def2‐TZVP basis set from EMSL Basis Set Exchange Library.^[^ ^45^, ^46^ ^]^ All the optimized geometries were characterized as true minima or transition states by vibrational analysis. The compositions of the molecular orbitals were calculated using the AOMix program.^[^ ^47^ ^]^ The natural atomic orbital (NAO) charges and Wiberg bond indices were computed with the NBO 6.0 program.^[^ ^48^ ^]^ The UV‐visible transitions were calculated on the above‐mentioned optimized geometries by means of time‐dependent DFT (TD‐DFT) calculations, with the CAM‐B3LYP functional^[^ ^49^ ^]^ and the Def2‐TZVP basis set. The UV–visible spectra were simulated from the computed TD‐DFT transition energies and their oscillator strengths by using the SWizard program,^[^ ^50^ ^]^ each transition being associated with a Gaussian function of half‐height width equal to 2000 cm^−1^.

[CCDC 2410767 (for **RhH_2_Ag_19_ *_o_***) and 2410768 (for **RhH_2_Ag_19_ *_o_*_N_**) contain the supplementary crystallographic data for this paper. These data can be obtained free of charge from The Cambridge Crystallographic Data Centre via ww.ccdc.cam.ac.uk/data_request/cif].

## Conflict of Interest

The authors declare no conflict of interest.

## Supporting information



Supporting Information

Supporting Information

## Data Availability

The data that support the findings of this study are available in the supplementary material of this article.
