# Supplementary material for: Hydride Migration within RhH2Ag19 Superatom: A Combined Neutron Diffraction and DFT Analysis
Source: Small. 2025 Mar 23;21(18):2501583. doi: 10.1002/smll.202501583 (PMC12051829; doi:10.1002/smll.202501583)
Supplement: Supplementary file 1 — Supporting Information [file SMLL-21-2501583-s001.docx]

Supporting Information

Hydride Migration within RhH_2_Ag_19_ Superatom: A Combined Neutron Diffraction and DFT Analysis

Tzu-Hao Chiu,^a^ Michael N. Pillay,^a^ Jian-Hong Liao,^a^ Xiaoping Wang,^b^ Hao Liang,^c^ Samia Kahlal,^c^ Jean-Yves Saillard,^c^* and C. W. Liu^a^*

**
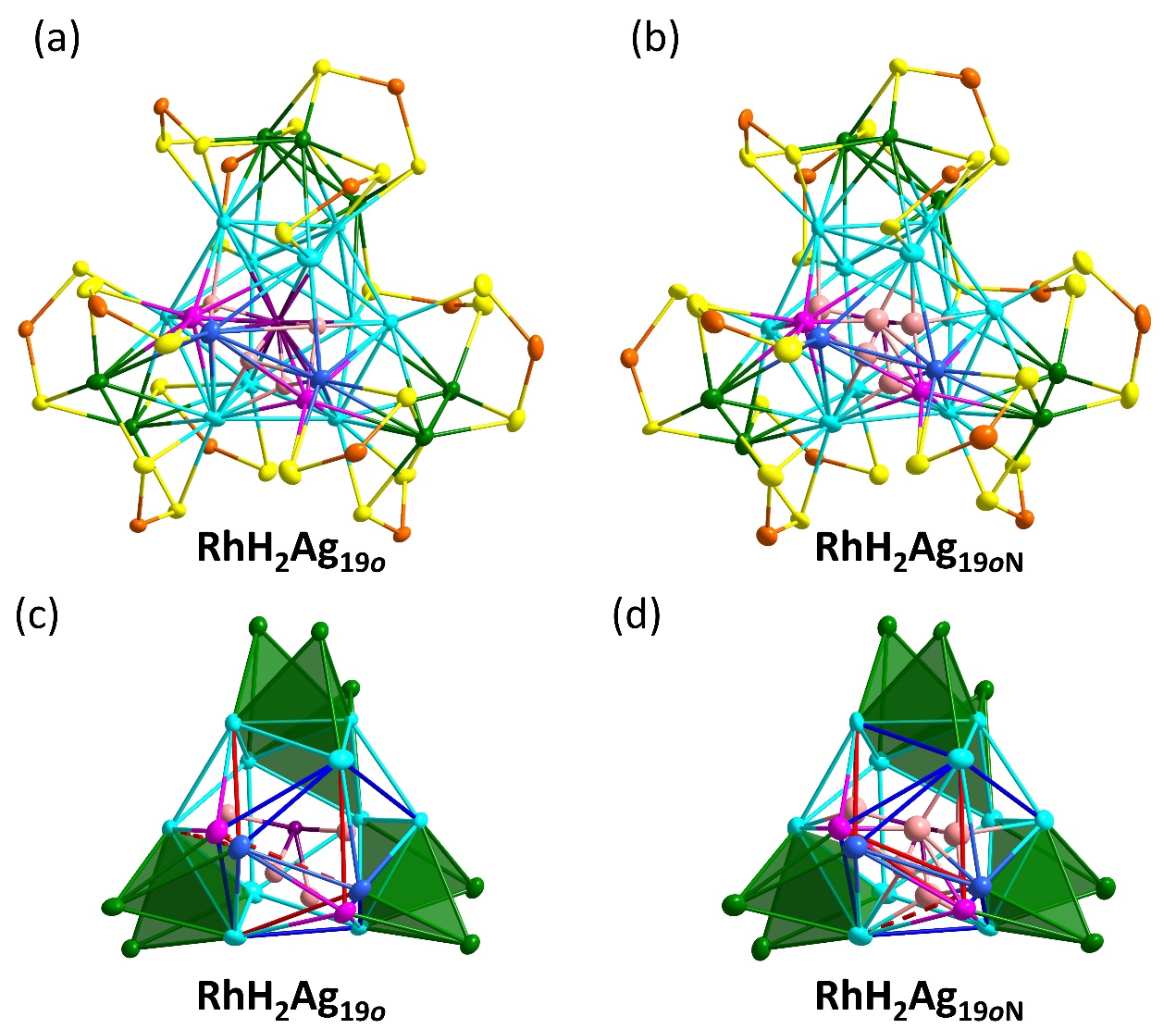
**

**Figure S1.** Total structure of (a) **RhH_2_Ag_19_*_o_*** and (b) **RhH_2_Ag_19_*_o_*_N_**. Metal-framework of (c) **RhH_2_Ag_19_*_o_*** (d) **RhH_2_Ag_19_*_o_*_N_**. Color code: sky blue for Ag_ico_, blue for Ag_ico_ with 55% occupancy, magenta for Ag_ico_ with 45% occupancy, purple for Rh, pink for H, yellow for S, orange for P.

**Figure S2.** VT ^1^H NMR spectrum (THF-*d*_8_) of **RhH_2_Ag_19_** ^S1^ Broadening of the hydride resonance at low temperature and a lack ^107/109^Ag coupling indicate hydride migration.


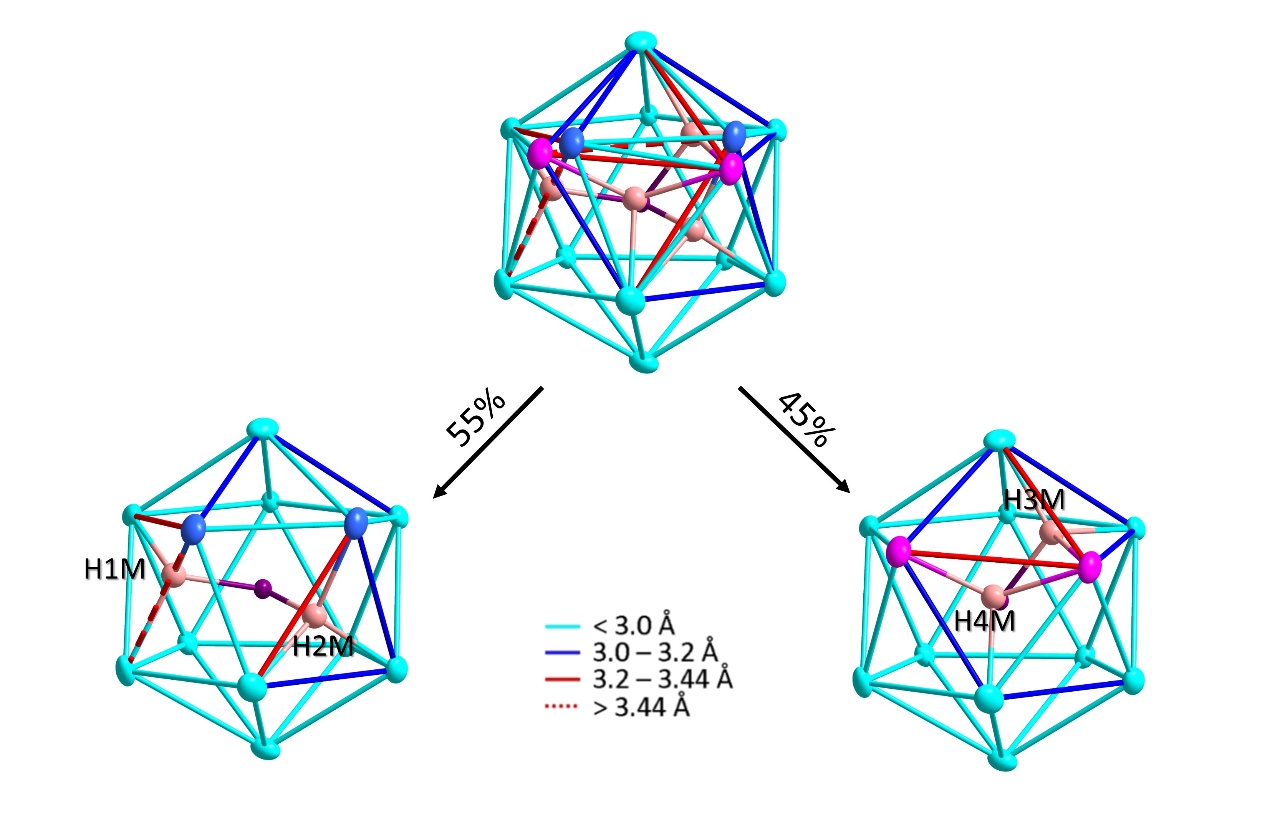


**Figure S3.** Possible positions of hydrides of **RhH_2_Ag_19_*_o_*** in the icosahedron. Color code: sky blue for Ag_ico_, blue for Ag_ico_ with 55% occupancy, magenta for Ag_ico_ with 45% occupancy, purple for Rh, pink for H.


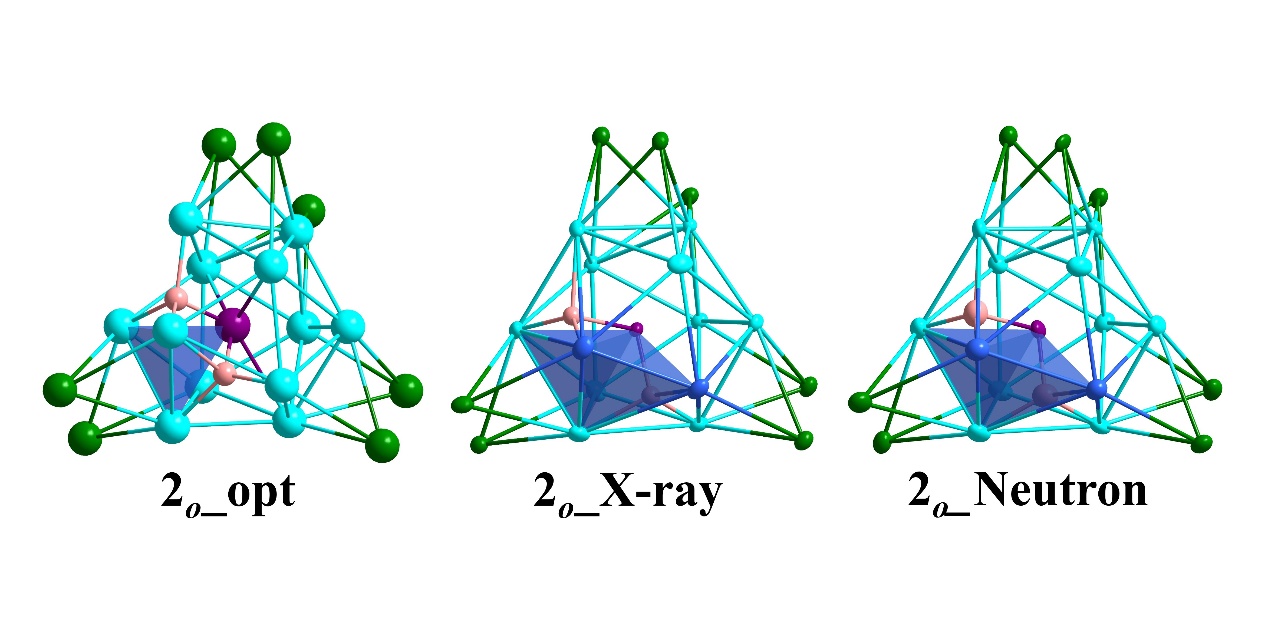


**Figure S4.** Solid-state and optimized structure of **2*_o_***

**
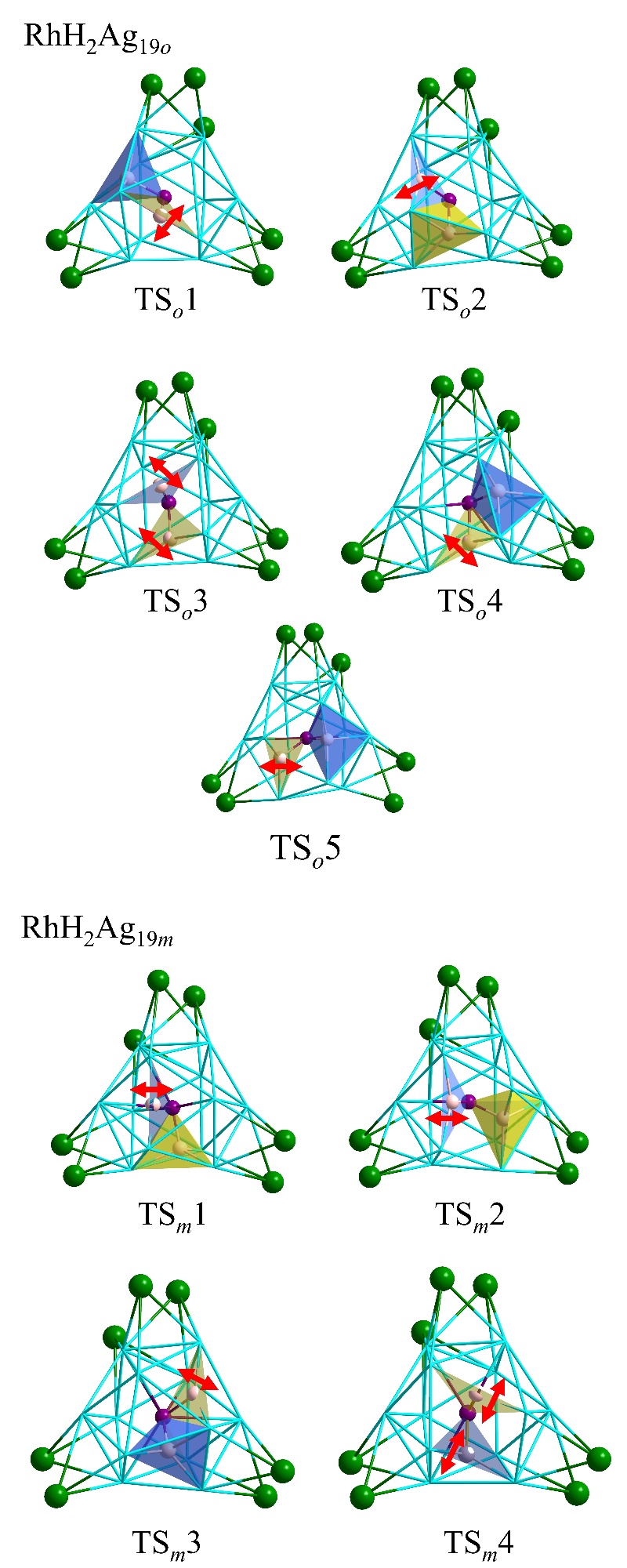
**

**Figure S5.** Oscillation directions of hydrides in each transition state


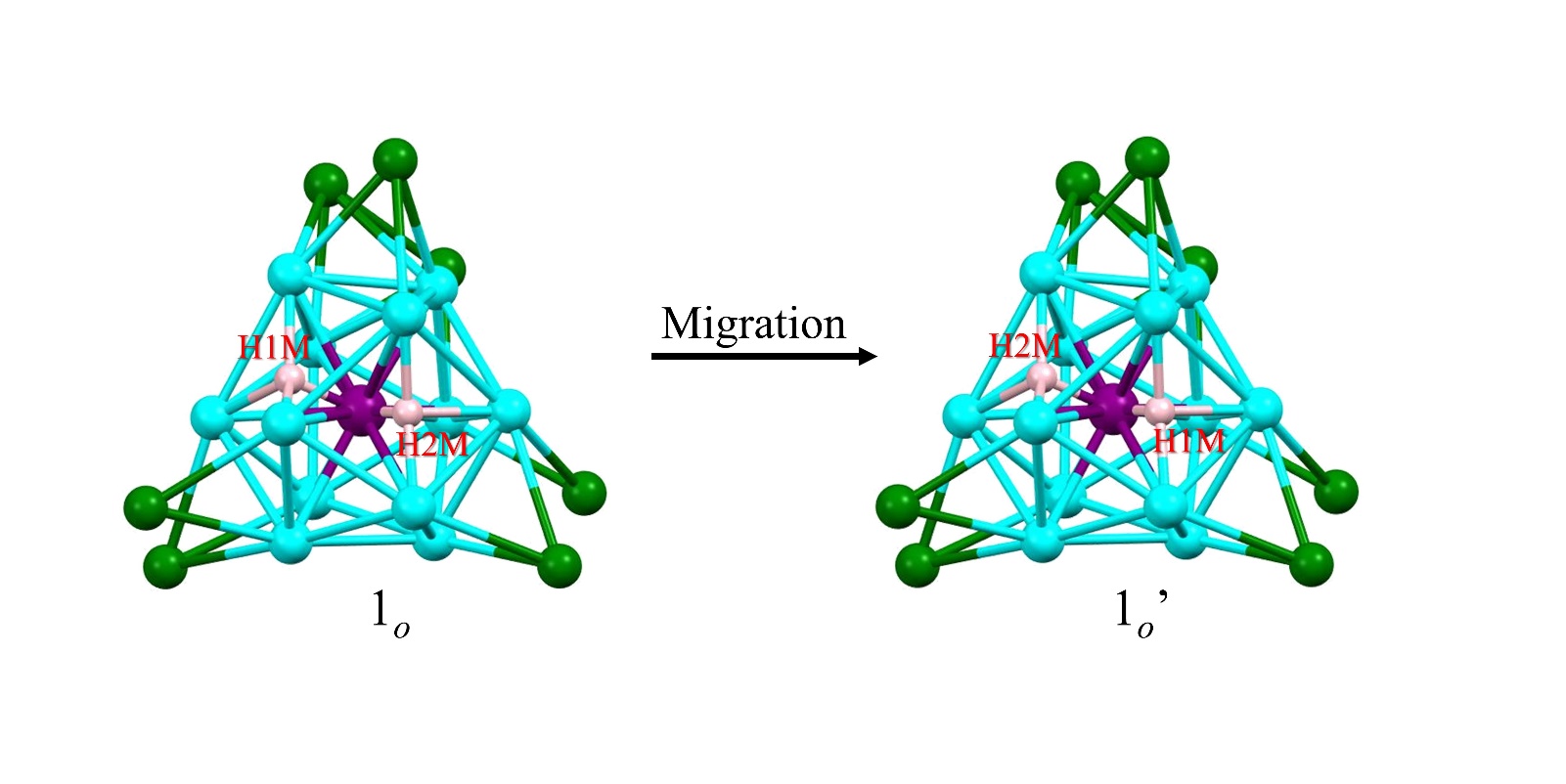


**Figure S6.** Isomer **RhH_2_Ag_19_*_o_*** before and after complete hydride migration


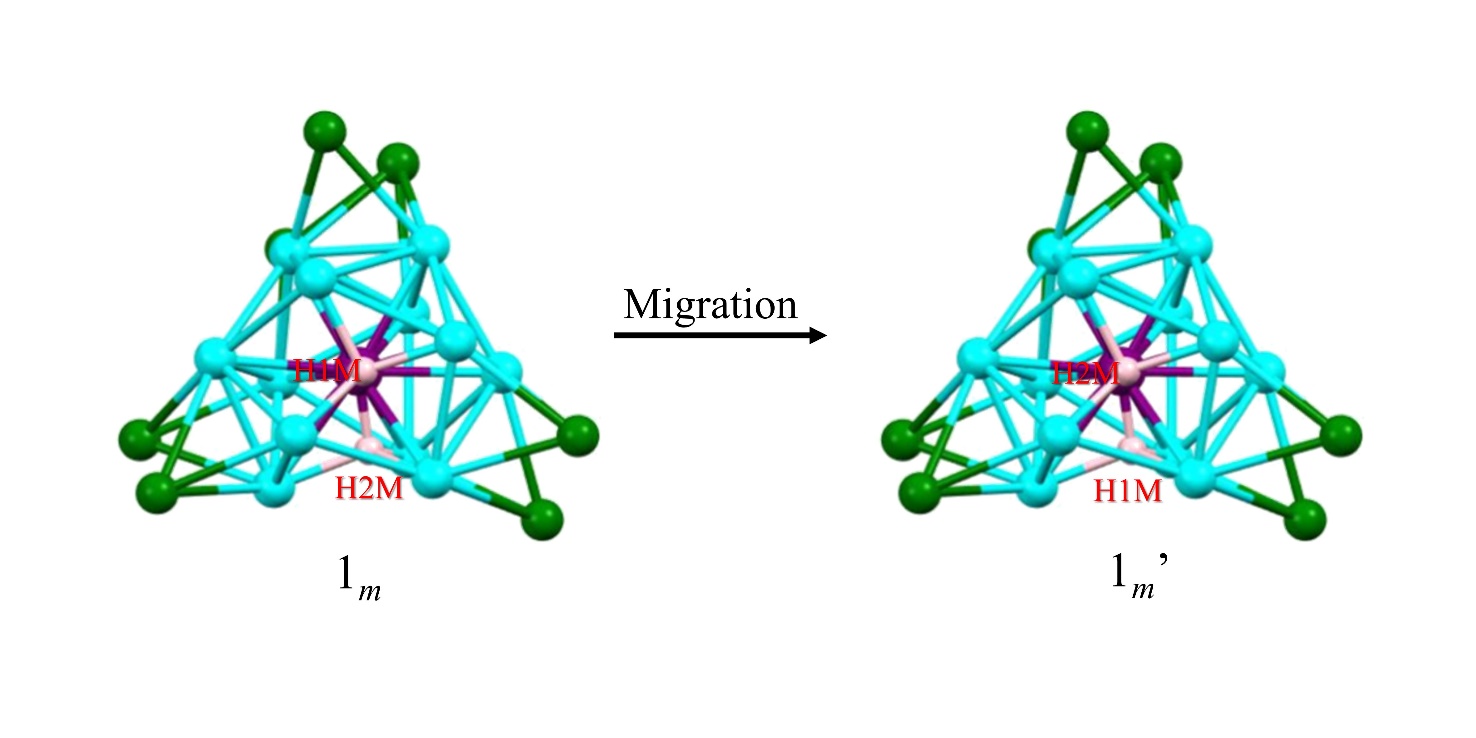


**Figure S7.** Isomer **RhH_2_Ag_19_*_m_*** before and after complete migration.

**
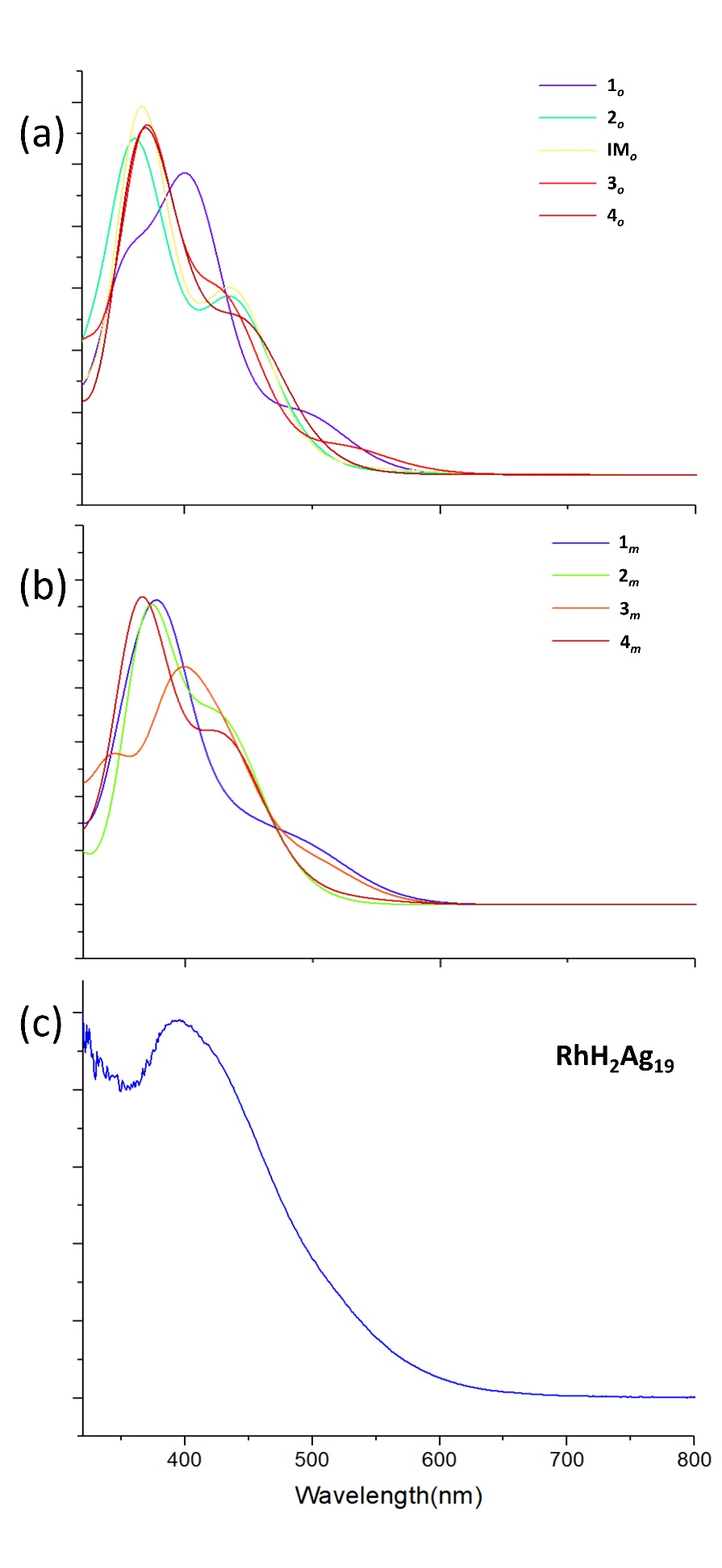
**

**Figure S8.** TD-DFT-simulated UV-vis absorption spectra of (a) **RhH_2_Ag_19_*_o_*** (b) **RhH_2_Ag_19_*_m_*** and absorption spectrum of **RhH_2_Ag_19_**.


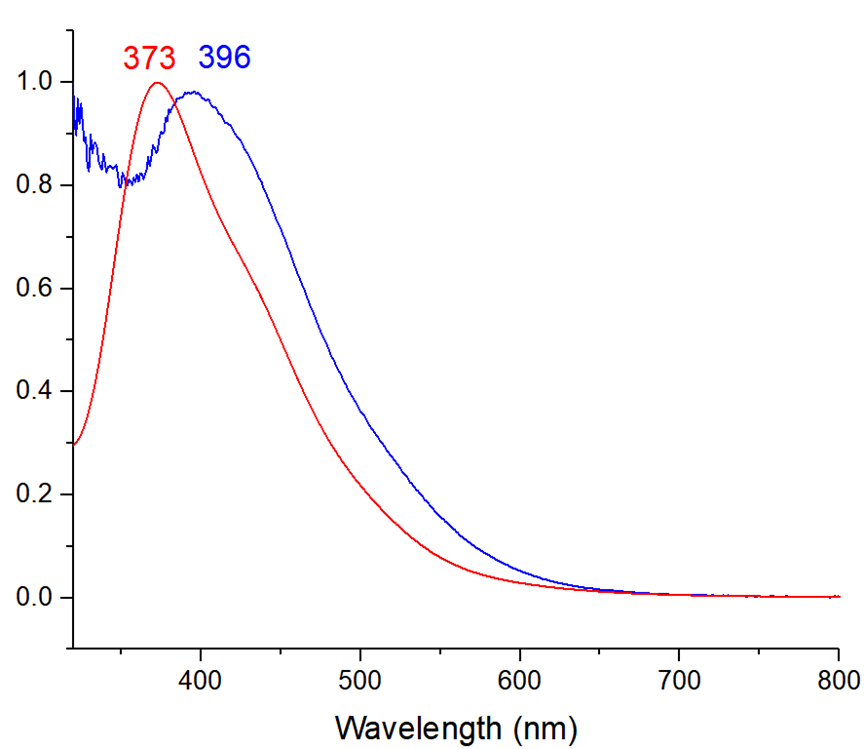


**Figure S9.** TD-DFT-simulated UV-vis absorption spectrum of **RhH_2_Ag_19EXP_** (blue) and **RhH_2_Ag_19DFT_** (red). (**RhH_2_Ag_19DFT_**= 2$\times$**1*_o_*** + 2$\times$**1*_m_*** + **2*_o_*** + **2*_m_*** + **IM*_o_*** + **3*_m_*** + **3*_o_*** + **4*_m_*** + **4*_o_***)


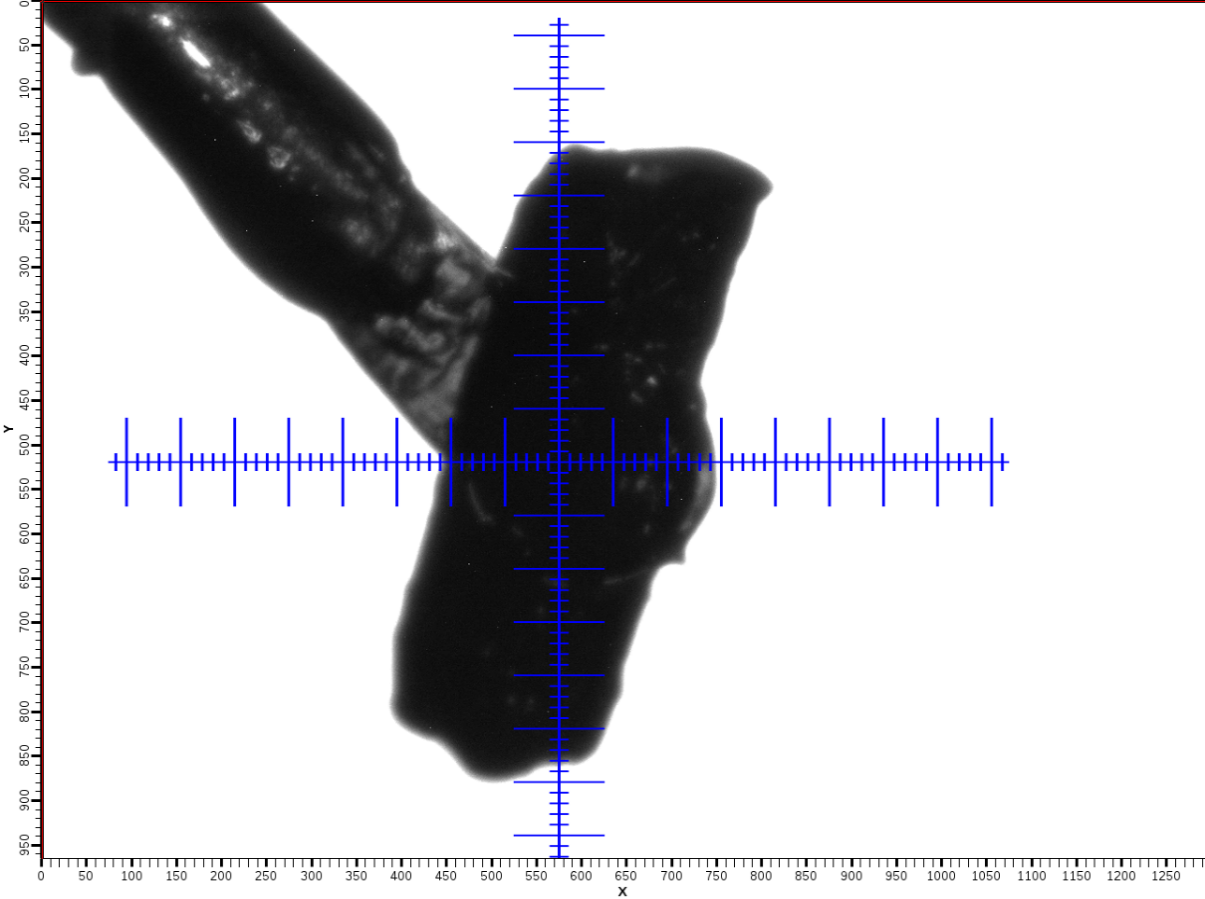


**Figure S10.** The single crystal image of **RhH_2_Ag_19_*_o_*_N_** on a camera in the TOPAZ instrument for neutron diffraction.


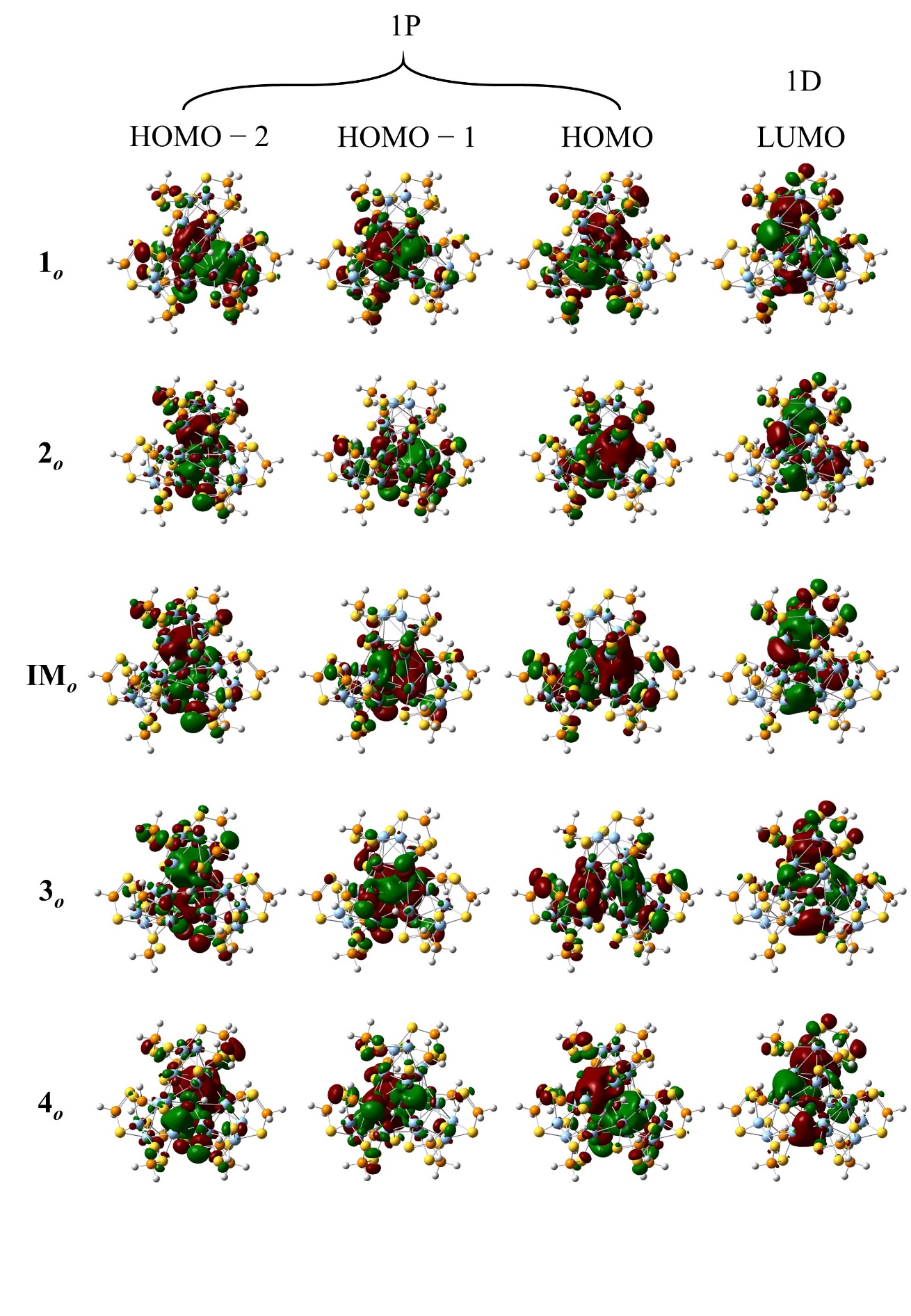


**Figure S11.** Kohn-Sham frontier orbitals of **RhH_2_Ag_19_*_o_***


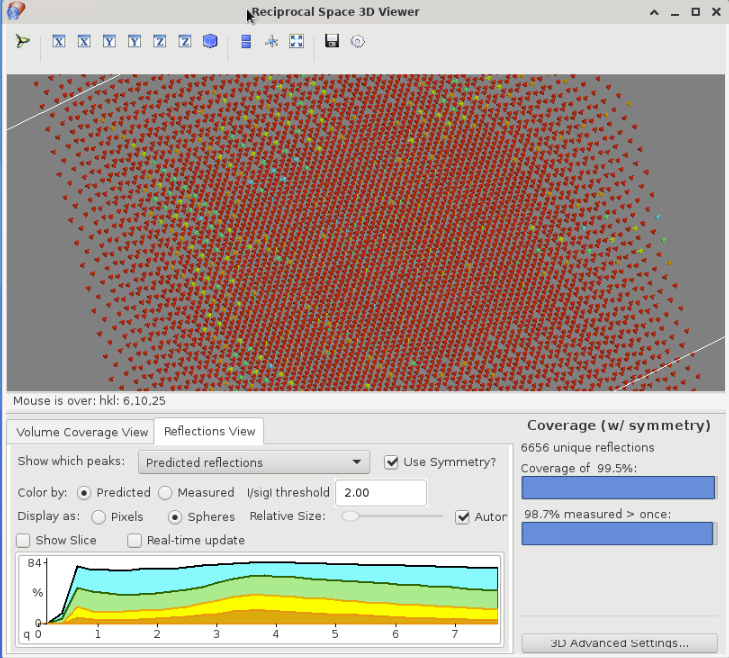


**Figure S12.** Reflection coverage of **RhH_2_Ag_19_*_o_*_N_**

**Table S1.** Selected experimental angles (deg.) for **2*_o_*** and **4*_o_***

| **Compound** | **2*_o_*** | **4*_o_*** |
| --- | --- | --- |
| X-ray  ∠HRhH | 102 | 69 |
| opt ∠HRhH | 87 | 81 |
| **ΔE** | 0.13eV | 0.13eV |
| Frozen_opt ∠HRhH | 98 | 71 |
| **ΔE** | 0eV | 0.18eV |
| **ΔE_o-froz_** | 3.4eV | 3.6eV |

**Table S2.** Selected experimental angles (deg.) for **1*_o_***, **2*_o_***, **3*_o_*** and **4*_o_***

| **Compound** | **1*_o_*** | **2*_o_*** | **3*_o_*** | **4*_o_*** |
| --- | --- | --- | --- | --- |
| Neutron  ∠HRhH | 93 | 96 | 66 | 46 |
| opt ∠HRhH | 94 | 87 | 84 | 81 |
| **ΔE** | 0eV | 0.13eV | 0.15eV | 0.13eV |
| Frozen_opt ∠HRhH | 99 | 97 | 73 | 72 |
| **ΔE** | 0eV | 0.01eV | 0.02eV | 0.15eV |
| **ΔE_o-froz_** | 14eV | 14eV | 14eV | 14eV |

**Table S3.** Selected distances (Å) and angles (deg.) for DFT-Optimized structure **1*_o_***, **2*_o_***, **IM*_o_***, **3*_o_*** and **4*_o_***

| Comp. | 1*_o_* | 2*_o_* | IM*_o_* | 3*_o_* | 4*_o_* |
| --- | --- | --- | --- | --- | --- |
| CSM | 0.54 | 0.46 | 0.5 | 0.45 | 0.53 |
| Rh–Ag_ico_ | 2.849–3.044  avg. 2.912 | 2.828–3.099  avg. 2.916 | 2.831–3.051  avg. 2.907 | 2.849–2.989  avg. 2.918 | 2.808–2.982  avg. 2.919 |
| Ag_ico_–Ag_ico_ | 2.890–3.710  avg. 3.040 | 2.912–3.666  avg. 3.063 | 2.885–3.733  avg. 3.085 | 2.885–3.592  avg. 3.087 | 2.898 –3.706  avg. 3.090 |
| Ag_ico_–Ag_cap_ | 3.005–3.242  avg. 3.147 | 3.015–3.295  avg. 3.135 | 3.013–3.224  avg. 3.131 | 3.021–3.218  avg. 3.124 | 3.023–3.301  avg. 3.137 |
| Rh–H | 1.671–1.686  avg. 1.679 | 1.663–1.666  Avg. 1.665 | 1.660–1.675  avg. 1.668 | 1.652–1.680  Avg. 1.666 | 1.659–1.668  Avg. 1.664 |
| Ag_ico_–H | 1.973–2.209  avg. 2.056 | 1.949–2.192  avg. 2.062 | 1.976–2.264  avg. 2.063 | 1.991–2.146  avg. 2.067 | 1.971–2.231  avg. 2.062 |
| H–Rh–H | 94 | 87 | 88 | 84 | 81 |

**Table S4.** Selected experimental distances (Å) and angles (deg.) for DFT-Optimized structure **1*_m_***, **2*_m_***, **3*_m_*** and **4*_m_***

| Comp. | 1*_m_* | 2*_m_* | 3*_m_* | 4*_m_* |
| --- | --- | --- | --- | --- |
| CSM | 0.47 | 0.58 | 0.51 | 0.44 |
| Rh–Ag_ico_ | 2.852–3.003  avg. 2.914 | 2.817–2.982  avg. 2.921 | 2.818–3.028  avg. 2.916 | 2.832–3.009  avg. 2.920 |
| Ag_ico_–Ag_ico_ | 2.915–3.717  avg. 3.064 | 2.902–3.757  avg. 3.066 | 2.893–3.683  avg. 3.064 | 2.918–3.607  avg. 3.065 |
| Ag_ico_–Ag_cap_ | 3.029–3.265  avg. 3.130 | 3.039–3.277  avg. 3.136 | 3.017–3.290  avg. 3.144 | 2.996–3.255  avg. 3.133 |
| Rh–H | 1.674–1.685  avg. 1.680 | 1.664–1.664  Avg. 1.664 | 1.673–1.678  avg. 1.676 | 1.658–1.663  Avg. 1.661 |
| Ag_ico_–H | 1.953–2.128  avg. 2.032 | 1.975–2.211  avg. 2.064 | 1.953–2.266  avg. 2.059 | 1.964–2.225  avg. 2.056 |
| H–Rh–H | 104 | 81 | 91 | 82 |

**Table S5.** Selected X-ray crystallographic data of **RhH_2_Ag_19_*_o_***.

| Compound | **RhH_2_Ag_19_*_o_*** |
| --- | --- |
| CCDC no. | 2410767 |
| Chemical formula | C_72_H_170_Ag_19_O_24_P_12_RhS_24_ |
| Formula weight | 4713.59 |
| Wavelength, Å | 0.71073 |
| Crystal System | Orthorhombic |
| Space group | *Pbca* |
| a, Å | 27.6310(18) |
| b, Å | 28.7484(17) |
| c, Å | 35.688(2) |
| α, deg. | 90 |
| β, deg. | 90 |
| γ, deg. | 90 |
| V, Å^3^ | 28349(3) |
| Z | 8 |
| Temperature, K | 100(2) |
| ρ_calcd_, g/cm^3^ | 2.209 |
| μ, mm^-1^ | 3.212 |
| θ_max_, deg. | 25.000 |
| Completeness, % | 100 |
| Reflection collected / unique | 217580 / 24953  [R(int) = 0.1047] |
| Restraints / parameters | 431 / 1440 |
| ^a^ *R*1, ^b^ *wR*2 [I > 2σ(I)] | 0.0376, 0.0668 |
| ^a^ *R*1, ^b^ *wR*2 (all data) | 0.0599, 0.0769 |
| GOF | 1.039 |
| Largest diff. peak and hole, e/Å^3^ | 2.407 and -2.125 |

*^a^ R*1 = ∑∥*F_o_*| − |*F_c_*∥/ ∑|*F_o_*|. *^b^* *wR*2 = {∑[*w*(*F_o_*^2^ − *F_c_*^2^)^2^] / ∑[*w*(*F_o_*^2^)^2^ ]}^1/2^.

**Table S6.** Selected neutron crystallographic data of **RhH_2_Ag_19_*_o_*_N_**

| Compound | **RhH_2_Ag_19_*_o_*_N_** |
| --- | --- |
| CCDC no. | 2410768 |
| formula | C_72_H_170_Ag_19_O_24_P_12_RhS_24_ |
| formula weight | 4713.59 |
| crystal system | Orthorhombic |
| space group | *Pbca* |
| a (Å) | 27.6645(13) |
| b (Å) | 28.7724(17) |
| c (Å) | 35.7535(19) |
| α (deg) | 90 |
| β (deg) | 90 |
| γ (deg) | 90 |
| V (Å^3^) | 28459(3) |
| Z | 8 |
| T (K) | 100 |
| crystal size (mm) | 0.85 x1.06 x 2.95 |
| λ (Å) | 0.43-3.50 |
| ρ (Mg m^-3^) | 2.209 |
| μ (cm^-1^) | 0.2312 |
| measured reflns. / unique | 19939 / 6680 |
| restraints / no. of params. refined | 2202 / 1623 |
| *R*1*^a^* (observed), *wR*2*^b^* (all) | 0.100, 0.229 |
| GOF | 1.141 |
| largest diff. peak and hole (fm Å^-3^) | 1.26 and -1.18 |

*^a^ R*1 = ∑∥*F_o_*| − |*F_c_*∥/ ∑|*F_o_*|. *^b^* *wR*2 = {∑[*w*(*F_o_*^2^ − *F_c_*^2^)^2^] / ∑[*w*(*F_o_*^2^)^2^ ]}^1/2^.

**Table S7.** Atomic coordinates of the DFT-optimized structure of **1*_o_***-**4*_o_***, **1*_m_***-**4*_m_***, TS*_o_*1-TS*_o_*5 and TS*_m_*1-TS*_m_*4

**1*_o_***

Rh 0.205681 -0.009879 0.278297

Ag -1.314739 1.182407 -1.899474

Ag 1.319628 -0.124809 -2.450256

Ag 1.161092 2.405627 -0.999783

Ag -1.202639 2.393206 0.874563

Ag 1.792926 2.056124 1.852692

Ag 3.067325 0.264859 -0.129758

Ag 1.673237 -2.377735 -0.567994

Ag -1.025608 -2.537547 1.118933

Ag -1.034038 -0.105511 2.901585

Ag -2.653531 -0.167543 0.376134

Ag -1.015955 -1.686376 -1.716726

Ag 1.987030 -0.859672 2.443385

Ag -3.603811 -1.954794 2.708763

Ag -3.496283 -3.132691 -0.554757

Ag -3.927355 -0.531899 -2.320606

Ag 0.825632 4.883055 0.756400

Ag -1.448893 4.223116 -1.609513

Ag 4.467003 -2.267509 1.074002

Ag 4.122820 -1.685877 -2.369423

H 0.707692 -1.407676 1.044178

H 0.201701 0.899345 1.697534

S -1.214712 -0.477344 5.421446

S -4.199606 0.290547 3.780362

S 3.325769 -0.506312 4.608903

S 3.308115 -3.558841 2.953351

S 3.971955 3.249991 2.610785

S 0.845259 4.558684 3.308944

S -3.928216 3.548861 -1.927645

S -2.829142 1.022084 -4.101018

S -1.191159 -3.387944 -3.644295

S -4.632807 -2.958329 -2.925587

S -2.000050 -3.859584 3.451744

S -1.899811 -5.071081 0.189613

S 5.447758 -3.528338 -1.007419

S 2.223534 -4.880676 -0.469238

S 5.131127 0.675534 -2.166853

S 5.529408 0.111638 1.244045

S 2.691548 4.635364 -1.043350

S 0.292138 4.132999 -3.538472

S -1.218543 6.325806 -0.024869

S -3.013428 3.909763 1.848531

S -5.182437 0.505186 -0.231711

S -5.540023 -2.795535 1.110180

S 1.961681 0.534142 -4.849447

S 2.567313 -2.818341 -4.046994

P -3.110485 0.184155 5.488826

P 3.753426 -2.467046 4.609679

P 2.850113 4.533045 3.663715

P -3.864583 2.756129 -3.764804

P -3.101811 -3.372627 -4.228481

P -2.401468 -5.346285 2.132221

P 4.059067 -5.009596 -1.262135

P 6.363464 0.403280 -0.582211

P 1.751579 5.265044 -2.739305

P -2.390989 5.794669 1.566191

P -6.299273 -0.987064 0.574884

P 2.264897 -1.363104 -5.429019

H -3.165921 1.481858 6.064611

H -3.821006 -0.558596 6.472534

H 3.119263 -3.128868 5.697240

H 5.117608 -2.654563 4.969606

H 2.991021 4.314300 5.062011

H 3.376460 5.851393 3.565843

H -5.192778 2.569576 -4.230830

H -3.367569 3.675439 -4.726410

H -3.418665 -4.630996 -4.805801

H -3.297222 -2.513033 -5.342499

H -3.760432 -5.748375 2.240726

H -1.738268 -6.516723 2.587009

H 4.655301 -6.208886 -0.789941

H 4.006167 -5.259298 -2.658966

H 7.215694 1.534330 -0.486305

H 7.307484 -0.622165 -0.860076

H 1.317215 6.605357 -2.552430

H 2.762476 5.442406 -3.719695

H -1.735811 6.301884 2.720553

H -3.537530 6.630189 1.513508

H -6.950479 -0.445108 1.712115

H -7.397565 -1.236948 -0.288217

H 1.182239 -1.825293 -6.225084

H 3.317030 -1.388513 -6.388026

**2*_o_***

Rh 0.266855 0.019118 0.249130

Ag -1.309817 0.709752 -2.090020

Ag 1.540117 0.079601 -2.440359

Ag 0.672296 2.538110 -0.971625

Ag -1.819010 2.039155 0.577764

Ag 0.594992 2.432369 2.165355

Ag 3.047345 0.892712 -0.083523

Ag 2.264185 -1.979068 -0.450981

Ag -0.519566 -2.572017 1.361524

Ag -1.142649 0.066689 2.714771

Ag -2.513290 -0.760608 0.182947

Ag -0.413478 -2.025095 -1.617255

Ag 1.817581 -0.367693 2.686499

Ag -3.338715 -2.357981 2.676083

Ag -2.612395 -3.875759 -0.489569

Ag -3.412791 -1.576320 -2.576859

Ag -0.446907 4.929040 0.610117

Ag -2.137946 3.665027 -2.021941

Ag 4.722629 -1.330390 1.465536

Ag 4.633737 -0.778090 -2.099237

H 0.972886 -1.247009 1.069241

H 1.332444 1.011536 1.053213

S -1.750985 0.021519 5.206815

S -4.561889 -0.168709 3.131344

S 3.088654 0.200665 4.832125

S 3.622065 -2.829499 3.214219

S 2.684347 3.914714 3.012492

S -0.658354 4.728668 3.176266

S -4.337941 2.421653 -2.567398

S -2.501936 0.055484 -4.391858

S -0.004547 -4.014270 -3.200406

S -3.519389 -4.143986 -2.955401

S -1.477865 -3.793908 3.753216

S -0.750261 -5.290195 0.695654

S 6.133148 -2.297757 -0.530716

S 3.254505 -4.330627 -0.296863

S 5.167439 1.742793 -1.733151

S 5.204850 1.221791 1.700816

S 1.681553 5.011135 -0.897209

S -0.182020 4.011837 -3.694687

S -2.601866 5.821148 -0.561063

S -4.035359 3.158341 1.299632

S -5.058944 -0.696532 -0.718826

S -4.814471 -3.824449 0.996206

S 2.201968 1.024680 -4.748696

S 3.647099 -2.090437 -4.039275

P -3.685577 0.431872 4.862273

P 3.741795 -1.697600 4.899870

P 1.267069 5.088796 3.775008

P -3.896289 1.550668 -4.315149

P -1.785616 -4.355293 -4.032800

P -1.445142 -5.455773 2.593165

P 5.135358 -4.042046 -0.919414

P 6.216508 1.668781 -0.000060

P 0.885171 5.419030 -2.734118

P -3.911689 5.103802 0.842651

P -5.897984 -2.342029 0.127725

P 3.213456 -0.603306 -5.347109

H -3.934450 1.827767 4.951493

H -4.446790 -0.035237 5.969820

H 3.072252 -2.437091 5.912859

H 5.068671 -1.721786 5.412344

H 1.229763 5.037367 5.195440

H 1.562148 6.467843 3.583582

H -5.096258 1.062324 -4.897503

H -3.483527 2.491163 -5.296031

H -1.809493 -5.678820 -4.547379

H -1.966693 -3.604126 -5.225303

H -2.718429 -6.087307 2.596616

H -0.658525 -6.434352 3.255329

H 5.937731 -5.094936 -0.405809

H 5.260922 -4.257780 -2.317759

H 6.847790 2.926895 0.185147

H 7.347424 0.819911 -0.143312

H 0.167922 6.643034 -2.648722

H 1.967544 5.797249 -3.570623

H -3.694321 5.889101 2.006673

H -5.196032 5.564907 0.447920

H -6.831002 -1.876371 1.089059

H -6.753959 -2.953038 -0.825537

H 2.541324 -1.236436 -6.428716

H 4.402518 -0.186996 -6.009036

**IM*_o_***

Rh 0.237553 0.087504 0.271106

Ag -1.384824 0.497042 -2.104887

Ag 1.490215 0.109457 -2.410557

Ag 0.363889 2.570763 -1.082122

Ag -2.085126 1.854226 0.513867

Ag 0.312971 2.593090 2.011097

Ag 2.980780 1.180200 -0.184925

Ag 2.364409 -1.740715 -0.185617

Ag -0.106158 -2.611638 1.356085

Ag -1.183077 0.131445 2.759429

Ag -2.374342 -1.030978 0.209961

Ag -0.205013 -2.138278 -1.552411

Ag 2.124939 -0.012477 2.503229

Ag -2.954678 -2.511958 2.862027

Ag -2.184797 -4.144933 -0.211784

Ag -3.238944 -2.067433 -2.483705

Ag -1.037019 4.885783 0.443539

Ag -2.546905 3.325606 -2.147593

Ag 4.955066 -0.722605 1.354083

Ag 4.552399 -0.579928 -2.165831

H 0.454854 -0.724310 1.719655

H 1.235544 1.273336 0.865342

S -1.548586 0.150754 5.298421

S -4.385616 -0.458414 3.357736

S 3.286351 1.045921 4.555913

S 4.141467 -2.094113 3.360358

S 2.195410 4.332187 2.803205

S -1.217292 4.771611 3.014782

S -4.611299 1.828631 -2.567044

S -2.558129 -0.391387 -4.343220

S 0.186465 -3.709381 -3.563685

S -3.101929 -4.661479 -2.598340

S -0.932719 -3.669985 3.972127

S -0.176097 -5.329078 1.008236

S 6.294426 -1.814483 -0.618026

S 3.595145 -3.988024 0.085073

S 4.861681 2.008810 -2.129944

S 5.228231 1.885429 1.321731

S 1.079742 5.149504 -1.068725

S -0.627321 3.826225 -3.824225

S -3.249528 5.486237 -0.791365

S -4.391232 2.805314 1.238884

S -4.921272 -1.263586 -0.581178

S -4.335633 -4.213966 1.348062

S 2.017836 0.899938 -4.810706

S 3.494712 -2.135406 -3.889610

P -3.540974 0.228087 5.073465

P 4.188517 -0.720274 4.862340

P 0.662821 5.348337 3.575284

P -4.111985 0.937119 -4.289713

P -1.391662 -4.924158 -3.706863

P -0.806091 -5.402406 2.939595

P 5.395543 -3.653150 -0.719667

P 6.045701 2.245238 -0.501093

P 0.232981 5.398259 -2.909882

P -4.513750 4.703206 0.620836

P -5.580894 -2.897478 0.428218

P 2.899274 -0.829108 -5.323185

H -4.022913 1.555339 5.230365

H -4.153631 -0.398161 6.194518

H 3.666543 -1.377946 6.010069

H 5.529751 -0.510938 5.289144

H 0.659993 5.312992 4.996601

H 0.807289 6.747923 3.361545

H -5.261993 0.290810 -4.816586

H -3.837000 1.876137 -5.319220

H -1.013221 -6.274605 -3.478048

H -1.826167 -5.002779 -5.057384

H -2.023577 -6.131104 3.020796

H 0.075585 -6.272436 3.632538

H 6.314716 -4.580595 -0.161192

H 5.431867 -4.023898 -2.090433

H 6.538021 3.577113 -0.503892

H 7.254418 1.513769 -0.656308

H -0.651497 6.509287 -2.853169

H 1.246685 5.903487 -3.765473

H -4.456736 5.600105 1.721384

H -5.827399 4.956933 0.142671

H -6.505074 -2.435865 1.400158

H -6.424837 -3.651628 -0.428520

H 2.064524 -1.579183 -6.195379

H 3.987333 -0.550103 -6.197000

**3*_o_***

Rh 0.163000 0.246000 0.233000

Ag -1.661000 0.311000 -2.034000

Ag 1.246000 0.795000 -2.382000

Ag -0.576000 2.786000 -1.024000

Ag -2.491000 1.251000 0.739000

Ag -0.170000 2.409000 2.163000

Ag 2.659000 1.804000 0.033000

Ag 2.692000 -1.077000 -0.505000

Ag 0.619000 -2.405000 1.171000

Ag -1.161000 -0.475000 2.814000

Ag -2.036000 -1.577000 0.201000

Ag 0.210000 -1.904000 -1.745000

Ag 2.287000 -0.183000 2.283000

Ag -1.905000 -3.503000 2.622000

Ag -1.007000 -4.429000 -0.525000

Ag -2.816000 -2.550000 -2.551000

Ag -2.265000 4.421000 1.023000

Ag -3.606000 2.731000 -1.709000

Ag 5.153000 0.227000 1.106000

Ag 4.432000 0.887000 -2.304000

H 0.403000 0.344000 1.893000

H 0.662000 1.821000 0.273000

S -0.805000 -0.791000 5.419000

S -3.594000 -1.891000 3.690000

S 3.120000 0.979000 4.538000

S 4.719000 -1.591000 2.869000

S 1.238000 4.460000 3.073000

S -2.150000 3.991000 3.550000

S -5.231000 0.835000 -2.324000

S -2.711000 -0.628000 -4.296000

S 0.841000 -3.065000 -3.965000

S -1.965000 -4.974000 -2.913000

S 0.505000 -4.039000 3.421000

S 1.366000 -5.178000 0.234000

S 6.522000 -0.153000 -1.081000

S 4.423000 -2.984000 -0.755000

S 4.144000 3.424000 -1.808000

S 4.757000 2.784000 1.549000

S -0.494000 5.506000 -0.561000

S -1.932000 3.956000 -3.315000

S -4.654000 4.501000 -0.042000

S -4.896000 1.415000 1.682000

S -4.482000 -2.412000 -0.473000

S -2.992000 -5.295000 1.036000

S 1.420000 2.180000 -4.564000

S 3.597000 -0.532000 -4.254000

P -2.676000 -1.482000 5.470000

P 4.462000 -0.498000 4.579000

P -0.427000 4.969000 4.050000

P -4.542000 0.265000 -4.116000

P -0.331000 -4.674000 -4.121000

P 1.092000 -5.553000 2.196000

P 6.051000 -2.103000 -1.510000

P 5.346000 3.648000 -0.192000

P -1.562000 5.653000 -2.283000

P -5.562000 3.263000 1.314000

P -4.605000 -4.265000 0.347000

P 2.708000 0.886000 -5.401000

H -3.557000 -0.592000 6.142000

H -2.780000 -2.621000 6.317000

H 4.178000 -1.450000 5.596000

H 5.728000 -0.028000 5.028000

H -0.292000 4.794000 5.454000

H -0.649000 6.373000 3.993000

H -5.503000 -0.594000 -4.714000

H -4.525000 1.337000 -5.048000

H 0.426000 -5.871000 -4.010000

H -0.811000 -4.794000 -5.454000

H 0.236000 -6.673000 2.382000

H 2.313000 -6.061000 2.712000

H 7.189000 -2.875000 -1.154000

H 6.090000 -2.197000 -2.927000

H 5.510000 5.038000 0.045000

H 6.680000 3.272000 -0.504000

H -2.765000 6.373000 -2.047000

H -0.877000 6.548000 -3.145000

H -5.661000 4.018000 2.514000

H -6.928000 3.202000 0.928000

H -5.547000 -4.185000 1.404000

H -5.279000 -5.119000 -0.565000

H 3.698000 1.611000 -6.122000

H 2.088000 0.183000 -6.470000

**4*_o_***

Rh 0.176201 0.118948 0.322535

Ag -1.656964 0.916194 -1.814217

Ag 1.251593 0.693459 -2.380394

Ag 0.248548 2.827585 -0.670549

Ag -2.093804 1.875195 0.964519

Ag 0.720301 2.277642 2.204724

Ag 2.817766 1.336542 0.021996

Ag 2.298864 -1.541722 -0.761461

Ag 0.072535 -2.760549 0.800189

Ag -0.710092 -0.902610 2.929808

Ag -2.363539 -1.077884 0.310742

Ag -0.391478 -1.689800 -1.899160

Ag 2.150688 -1.140835 2.168146

Ag -2.581841 -3.367229 2.367344

Ag -2.137940 -4.010950 -0.937001

Ag -3.513634 -1.519447 -2.449498

Ag -0.933433 4.802415 1.333115

Ag -2.771444 3.758987 -1.332071

Ag 4.989307 -0.984372 0.914480

Ag 4.408461 0.189951 -2.366671

H 1.457039 0.535968 1.291291

H -0.669112 0.615303 1.672471

S -0.946227 -1.465770 5.404754

S -3.898715 -1.493709 3.533379

S 3.191862 -0.494408 4.562195

S 4.285390 -2.959505 2.405012

S 2.497647 3.792533 3.376278

S -0.913050 4.148238 3.820138

S -4.859078 2.318284 -1.814243

S -2.987973 0.433666 -4.098967

S 0.011381 -3.075860 -4.035991

S -3.328764 -3.970034 -3.290090

S -0.484800 -4.707838 3.030659

S 0.053466 -5.315429 -0.368630

S 6.265375 -1.379805 -1.334234

S 3.605114 -3.708782 -1.262891

S 4.661944 2.645527 -1.557597

S 5.167014 1.498347 1.678764

S 0.980749 5.410491 -0.328481

S -1.032421 4.568867 -3.068027

S -3.282672 5.611110 0.498286

S -4.327572 2.571959 1.995249

S -4.964512 -1.211610 -0.267629

S -4.197731 -4.588110 0.647699

S 1.603896 2.142976 -4.500832

S 3.296956 -0.908181 -4.379991

P -2.952902 -1.422885 5.330195

P 4.284597 -2.138090 4.281911

P 0.986793 4.647491 4.369488

P -4.515645 1.730419 -3.698834

P -1.795731 -3.685345 -4.625595

P -0.297421 -6.018389 1.502905

P 5.386811 -3.122674 -1.959106

P 5.907963 2.408944 0.031693

P -0.066756 5.964893 -1.989349

P -4.355927 4.572810 1.898123

P -5.513922 -3.072170 0.344737

P 2.646812 0.702290 -5.429914

H -3.459857 -0.266237 5.982480

H -3.469757 -2.437065 6.183745

H 3.901048 -3.194900 5.152664

H 5.628869 -1.943591 4.707106

H 1.060946 4.379674 5.763914

H 1.121504 6.064004 4.382011

H -5.713468 1.180232 -4.228373

H -4.314179 2.829308 -4.575817

H -1.668356 -4.912960 -5.328852

H -2.321722 -2.850193 -5.648091

H -1.398430 -6.917207 1.476351

H 0.764465 -6.905026 1.820059

H 6.326222 -4.158346 -1.710943

H 5.403609 -3.071631 -3.378679

H 6.373831 3.697228 0.403910

H 7.119227 1.796182 -0.389314

H -0.932298 7.037386 -1.641944

H 0.851468 6.624832 -2.847145

H -4.020296 5.150467 3.151840

H -5.705331 4.993047 1.759909

H -6.259472 -2.902482 1.539137

H -6.507696 -3.551256 -0.548033

H 1.906847 0.168956 -6.520493

H 3.736230 1.288086 -6.133938

**TS*_o_*1**

Rh 0.240763 -0.057291 0.246299

Ag -1.256925 0.895780 -2.037300

Ag 1.596586 0.247624 -2.393290

Ag 0.798980 2.556329 -0.775030

Ag -1.701111 2.059433 0.729984

Ag 0.814230 2.271766 2.216300

Ag 2.971331 0.778159 0.211697

Ag 2.302558 -1.995818 -0.707802

Ag -0.763140 -2.646571 1.253189

Ag -1.116047 -0.079555 2.711189

Ag -2.587811 -0.649600 0.124539

Ag -0.467488 -1.895583 -1.752193

Ag 1.759254 -1.207503 2.478769

Ag -3.560952 -2.355632 2.504223

Ag -2.806729 -3.683308 -0.786525

Ag -3.435883 -1.235738 -2.700377

Ag -0.206765 4.911314 0.897965

Ag -2.000150 3.880783 -1.737954

Ag 4.686173 -1.685156 1.351728

Ag 4.689637 -0.562802 -2.007701

H 0.823115 -1.571239 0.637226

H 1.121995 0.498664 1.549956

S -1.830276 -0.193191 5.147552

S -4.671521 -0.145553 3.081956

S 2.751910 -0.779975 4.872058

S 3.528795 -3.449236 2.818645

S 2.914023 3.529184 3.284594

S -0.377816 4.535396 3.441435

S -4.239527 2.759243 -2.364392

S -2.468408 0.505112 -4.387202

S -0.124130 -3.861416 -3.369206

S -3.645464 -3.756967 -3.297117

S -1.810676 -4.000354 3.487041

S -1.083895 -5.310195 0.346352

S 6.163709 -2.354643 -0.708402

S 3.261911 -4.362564 -0.848866

S 5.205227 1.862389 -1.278764

S 5.121589 0.788055 2.027027

S 1.894567 4.996968 -0.635693

S -0.076426 4.235456 -3.435200

S -2.362470 5.953262 -0.133612

S -3.864896 3.208390 1.543396

S -5.112117 -0.407434 -0.840601

S -5.030559 -3.643055 0.673038

S 2.269478 1.511993 -4.560653

S 3.705232 -1.640419 -4.101778

P -3.724944 0.381376 4.798150

P 3.573500 -2.590977 4.673500

P 1.559690 4.776496 4.052830

P -3.819315 2.027036 -4.180313

P -1.878960 -3.979287 -4.314692

P -1.868352 -5.564931 2.195916

P 5.155942 -4.000499 -1.390183

P 6.181711 1.527687 0.461822

P 1.082445 5.534714 -2.431222

P -3.644241 5.180602 1.266128

P -6.035341 -2.069939 -0.128535

P 3.363277 -0.013291 -5.266804

H -3.851036 1.793865 4.884543

H -4.509662 -0.017569 5.915472

H 2.980050 -3.541746 5.548732

H 4.907354 -2.599475 5.168582

H 1.514363 4.708933 5.472558

H 1.937429 6.137939 3.881902

H -5.031964 1.623782 -4.800237

H -3.379970 3.036988 -5.077046

H -1.962591 -5.237687 -4.967305

H -1.941207 -3.097376 -5.426913

H -3.191188 -6.077161 2.107468

H -1.198437 -6.656961 2.807467

H 5.937823 -5.132987 -1.041357

H 5.300047 -3.988304 -2.803155

H 6.763692 2.752824 0.880432

H 7.343643 0.740175 0.238682

H 0.433219 6.788834 -2.270751

H 2.167268 5.896277 -3.271718

H -3.327397 5.838357 2.485103

H -4.917603 5.742742 0.983127

H -6.982271 -1.634337 0.833459

H -6.882081 -2.589446 -1.142564

H 2.797760 -0.523396 -6.468082

H 4.599813 0.480024 -5.770085

**TS*_o_*2**

Rh 0.258022 0.085099 0.260141

Rh 0.258022 0.085099 0.260141

Ag -1.423679 0.514356 -2.065436

Ag 1.434841 -0.061430 -2.436723

Ag 0.496197 2.508957 -1.170623

Ag -1.935104 2.005206 0.532121

Ag 0.516686 2.645579 1.957613

Ag 3.039770 0.989321 -0.289356

Ag 2.268144 -1.889038 -0.197552

Ag -0.302242 -2.560446 1.445076

Ag -0.988700 0.214044 2.776299

Ag -2.452097 -0.852448 0.352316

Ag -0.376044 -2.146494 -1.472020

Ag 2.103357 0.117331 2.558167

Ag -3.076514 -2.321856 2.955162

Ag -2.431867 -4.052409 -0.140132

Ag -3.401703 -1.907338 -2.333369

Ag -0.734870 4.950372 0.273818

Ag -2.420940 3.400541 -2.174928

Ag 4.902062 -0.899734 1.360969

Ag 4.515420 -0.921386 -2.201262

H 0.773373 -0.880712 1.517696

H 1.303178 1.273612 0.757276

S -1.539179 0.262731 5.266716

S -4.420240 -0.190073 3.328771

S 3.449296 1.189865 4.469497

S 3.999857 -2.076845 3.447297

S 2.546518 4.362511 2.459607

S -0.821867 4.954874 2.860044

S -4.573970 2.022951 -2.574761

S -2.639180 -0.355147 -4.278179

S 0.049048 -4.020159 -3.192955

S -3.403420 -4.497975 -2.527453

S -1.097699 -3.590224 4.024930

S -0.451746 -5.287446 1.056257

S 6.190122 -2.152039 -0.543956

S 3.391069 -4.145832 0.280995

S 4.965762 1.637024 -2.298724

S 5.297942 1.687150 1.160504

S 1.346707 5.036052 -1.304406

S -0.532102 3.698174 -3.940696

S -2.971231 5.637742 -0.875506

S -4.211844 3.044183 1.204215

S -5.020110 -0.991574 -0.466164

S -4.551985 -3.973967 1.450043

S 2.048753 0.369737 -4.886534

S 3.490895 -2.636806 -3.777274

P -3.501073 0.576302 4.967892

P 4.196038 -0.632828 4.867025

P 1.112382 5.469150 3.288885

P -4.118701 1.058764 -4.268545

P -1.742909 -4.733105 -3.713979

P -1.058783 -5.331757 2.994319

P 5.199881 -3.943859 -0.552466

P 6.147836 1.891854 -0.674312

P 0.475444 5.244046 -3.140840

P -4.184695 4.961688 0.632845

P -5.741526 -2.612879 0.522040

P 2.730010 -1.484314 -5.258815

H -3.798256 1.965467 4.963657

H -4.195455 0.168938 6.140648

H 3.637659 -1.173903 6.057530

H 5.557116 -0.515775 5.264596

H 1.192163 5.477196 4.708260

H 1.295321 6.854622 3.019911

H -5.298553 0.460843 -4.786364

H -3.784013 1.950067 -5.322509

H -1.644833 -6.130254 -3.951552

H -2.129598 -4.276161 -5.002806

H -2.306021 -6.005233 3.097987

H -0.207074 -6.234326 3.683118

H 6.070563 -4.893170 0.045267

H 5.202183 -4.381604 -1.903897

H 6.723346 3.188087 -0.743277

H 7.309530 1.078649 -0.770901

H -0.312409 6.426905 -3.134101

H 1.506842 5.612313 -4.043859

H -3.941108 5.824066 1.735526

H -5.502927 5.352695 0.276077

H -6.663842 -2.130129 1.485251

H -6.595883 -3.332706 -0.353449

H 1.720362 -2.272444 -5.875260

H 3.668801 -1.398012 -6.324677

**TS*_o_*3**

Rh 0.155889 0.218184 0.286786

Ag -1.492040 0.287868 -2.125100

Ag 1.415870 0.527883 -2.308114

Ag -0.206908 2.703717 -1.051287

Ag -2.476161 1.454464 0.470423

Ag -0.300324 2.508760 2.114233

Ag 2.711692 1.704967 0.010900

Ag 2.662633 -1.223171 -0.225564

Ag 0.390391 -2.395923 1.402455

Ag -1.500804 -0.183661 2.749777

Ag -2.154081 -1.446055 0.164054

Ag 0.216647 -2.039163 -1.548676

Ag 2.174934 -0.067859 2.446380

Ag -2.366611 -3.213238 2.693832

Ag -1.244706 -4.408262 -0.359988

Ag -2.780372 -2.548947 -2.532193

Ag -2.009561 4.616432 0.591936

Ag -3.157152 2.872303 -2.170958

Ag 5.068461 0.093495 1.392083

Ag 4.590728 0.564367 -2.177704

H 0.286321 -0.114608 1.916542

H 0.820911 1.674538 0.684940

S -1.582973 -0.295403 5.372729

S -4.127624 -1.455717 3.327974

S 2.900390 1.191355 4.665285

S 4.487688 -1.551122 3.280231

S 1.203567 4.449269 3.139127

S -2.248193 4.341595 3.137603

S -4.872146 1.012510 -2.683385

S -2.404182 -0.781069 -4.409228

S 1.245383 -3.804241 -3.139711

S -2.040615 -5.019700 -2.826690

S -0.099376 -3.831609 3.762189

S 0.994231 -5.182110 0.737447

S 6.520981 -0.521195 -0.666379

S 4.275256 -3.220035 -0.244825

S 4.320824 3.132776 -1.764000

S 4.759699 2.687138 1.649409

S 0.018625 5.388339 -0.867441

S -1.286221 3.828181 -3.705374

S -4.252341 4.811119 -0.738152

S -4.951231 1.867688 1.110599

S -4.604869 -2.187383 -0.642668

S -3.375387 -5.059922 1.096163

S 1.827949 1.027187 -4.751041

S 4.846152 -0.739191 -4.366481

P -3.493970 -0.837356 5.169190

P 4.205694 -0.306525 4.877610

P -0.517013 5.181036 3.833134

P -4.179708 0.232001 -4.393569

P -0.358996 -4.678144 -3.949285

P 0.498705 -5.444483 2.677638

P 5.941919 -2.450143 -1.048380

P 5.469391 3.396194 -0.114109

P -0.829962 5.523402 -2.715574

P -5.399025 3.743828 0.582880

P -4.879781 -3.997972 0.235145

P 3.012208 -0.552427 -5.185342

H -4.385410 0.208844 5.530880

H -3.846264 -1.815390 6.140911

H 3.868806 -1.157140 5.966579

H 5.466690 0.177301 5.326322

H -0.603430 5.089533 5.249287

H -0.583966 6.593150 3.670265

H -5.179370 -0.615060 -4.941812

H -4.058978 1.205841 -5.420154

H 0.021124 -5.941370 -4.474850

H -0.788477 -4.015748 -5.130840

H -0.447169 -6.495753 2.820713

H 1.622543 -6.002210 3.340827

H 7.032921 -3.280324 -0.677594

H 5.939951 -2.577943 -2.463211

H 5.712060 4.787784 0.031603

H 6.786674 2.918709 -0.352120

H -1.950907 6.394509 -2.649781

H 0.054674 6.280235 -3.527524

H -5.538247 4.575208 1.726474

H -6.722045 3.779673 0.066622

H -5.898798 -3.831734 1.207401

H -5.514587 -4.861235 -0.695513

H 2.285619 -1.758999 -4.982504

H 3.169137 -0.567513 -6.597770

**TS*_o_*4**

Rh 0.118850 0.217274 0.244330

Ag -1.758637 0.541771 -1.969408

Ag 1.150870 0.891868 -2.372315

Ag -0.367688 2.841296 -0.743212

Ag -2.525793 1.396420 0.740882

Ag 0.049556 2.185860 2.450556

Ag 2.688102 1.778065 -0.026298

Ag 2.589755 -1.096216 -0.641668

Ag 0.613315 -2.555362 0.965283

Ag -0.610966 -0.825868 2.996645

Ag -2.089378 -1.496425 0.251738

Ag 0.016270 -1.775758 -1.855615

Ag 2.150243 -0.240702 2.192405

Ag -1.914193 -3.657555 2.442532

Ag -1.244804 -4.333164 -0.797540

Ag -3.104137 -2.230409 -2.484886

Ag -1.999966 4.436935 1.367328

Ag -3.349256 3.136969 -1.603554

Ag 5.074350 -0.044217 1.015866

Ag 4.396081 0.847341 -2.411190

H -0.849548 0.487134 1.592554

H 0.888590 1.604917 0.708197

S -0.720808 -1.427999 5.479989

S -3.566070 -2.033993 3.555182

S 3.212545 0.490699 4.466996

S 4.670243 -2.027869 2.569985

S 1.578768 4.128140 3.410690

S -1.828657 3.786989 3.842120

S -5.203418 1.405960 -2.153340

S -2.934162 -0.297450 -4.228061

S 0.673111 -3.060121 -3.989801

S -2.390017 -4.634385 -3.156399

S 0.436406 -4.444306 3.155597

S 1.146203 -5.162808 -0.188575

S 6.436229 -0.316921 -1.214824

S 4.206589 -3.061870 -1.097683

S 4.230662 3.349467 -1.721189

S 4.879900 2.508390 1.580041

S -0.160508 5.453241 -0.201282

S -1.721400 4.421409 -3.161127

S -4.354573 4.768055 0.248486

S -4.635735 1.784910 2.133817

S -4.607501 -2.148637 -0.309757

S -3.199472 -5.256101 0.767157

S 1.379414 2.271247 -4.535760

S 3.482808 -0.517704 -4.350145

P -2.691103 -1.798062 5.374778

P 4.424723 -1.102185 4.365643

P -0.065821 4.660206 4.409543

P -4.665759 0.752625 -3.967885

P -0.927442 -4.136219 -4.508318

P 0.941331 -5.789727 1.724533

P 5.898009 -2.219752 -1.753534

P 5.497118 3.397560 -0.132112

P -1.193768 5.900859 -1.905518

P -5.469415 3.377628 1.252270

P -4.788640 -4.057939 0.366517

P 2.540080 0.905836 -5.443362

H -3.435465 -0.777526 6.026086

H -3.005927 -2.907875 6.207432

H 4.021971 -2.119746 5.273332

H 5.694351 -0.770551 4.916985

H 0.052114 4.401433 5.802592

H -0.226191 6.073906 4.431945

H -5.735450 -0.018996 -4.494933

H -4.604369 1.811394 -4.912233

H -0.503610 -5.366009 -5.079574

H -1.606672 -3.556123 -5.613768

H 0.074158 -6.914688 1.782605

H 2.172519 -6.379080 2.112571

H 6.987243 -3.059278 -1.398426

H 5.982298 -2.251762 -3.171426

H 5.790229 4.758420 0.147943

H 6.779603 2.920442 -0.516335

H -2.311906 6.706320 -1.557496

H -0.404086 6.833599 -2.627453

H -6.224152 4.085672 2.224542

H -6.495198 2.987159 0.349763

H -5.592545 -3.999106 1.533393

H -5.633363 -4.765653 -0.527806

H 1.802810 0.200378 -6.432961

H 3.480744 1.582468 -6.269419

**TS*_o_*5**

Rh 0.239264 0.095348 0.255068

Ag -1.477842 1.093455 -1.869003

Ag 1.314432 0.313866 -2.468512

Ag 0.726686 2.717505 -0.872091

Ag -1.608150 2.166255 0.968012

Ag 1.404616 2.158736 1.961385

Ag 3.049361 0.948214 -0.222634

Ag 1.995564 -1.986972 -0.632054

Ag -0.462481 -2.634919 0.969787

Ag -1.027737 -0.436657 2.881811

Ag -2.528699 -0.607067 0.294959

Ag -0.676403 -1.715303 -1.830981

Ag 1.764708 -1.302961 2.419592

Ag -3.213859 -2.671867 2.487868

Ag -2.814025 -3.624860 -0.778137

Ag -3.716601 -1.004678 -2.461218

Ag 0.020707 4.908482 1.127886

Ag -2.103605 4.057588 -1.404934

Ag 4.694953 -1.740350 1.124338

Ag 4.389854 -0.796962 -2.374257

H 1.776831 -0.206116 0.789138

H -0.087082 0.857823 1.719486

S -1.366111 -0.817662 5.399229

S -4.216161 -0.498687 3.421474

S 2.839238 -0.798264 4.774225

S 3.663241 -3.454916 2.716847

S 3.303285 3.505258 3.023332

S 0.018360 4.456737 3.649657

S -4.420938 3.012269 -1.870644

S -2.885516 0.812403 -4.131856

S -0.499915 -3.241427 -3.909372

S -3.946671 -3.491965 -3.153991

S -1.350797 -4.276037 3.258377

S -0.899489 -5.248407 -0.064942

S 5.896379 -2.542594 -1.071642

S 2.885841 -4.376698 -0.863002

S 5.157983 1.616361 -1.766984

S 5.367753 0.721321 1.584368

S 1.925509 5.167675 -0.635192

S -0.298165 4.473862 -3.236434

S -2.214561 6.048445 0.331314

S -3.674456 3.274775 1.983550

S -5.125932 -0.349060 -0.333060

S -4.955069 -3.744243 0.795683

S 2.025453 1.371559 -4.708640

S 3.105891 -1.924621 -4.253055

P -3.323234 -0.409117 5.247674

P 3.772368 -2.550259 4.546835

P 1.997049 4.611394 4.075022

P -4.182332 2.346519 -3.743669

P -2.382440 -3.574933 -4.482143

P -1.396793 -5.722059 1.842466

P 4.753077 -4.169399 -1.555547

P 6.305386 1.249885 -0.123413

P 0.890423 5.786328 -2.276210

P -3.357439 5.247769 1.828037

P -5.996228 -2.063038 0.329846

P 2.666417 -0.367667 -5.477283

H -3.620672 0.886041 5.751511

H -4.047583 -1.211288 6.173220

H 3.307591 -3.521040 5.476255

H 5.133446 -2.454320 4.950632

H 2.116965 4.342782 5.465791

H 2.396014 5.976915 4.050493

H -5.453463 1.987865 -4.266345

H -3.806365 3.383668 -4.638199

H -2.470727 -4.854500 -5.093086

H -2.754441 -2.739549 -5.569945

H -2.648107 -6.396099 1.863844

H -0.524768 -6.761710 2.258435

H 5.497153 -5.318283 -1.177595

H 4.798220 -4.266667 -2.972046

H 7.069033 2.420012 0.125991

H 7.329565 0.322909 -0.457164

H 0.182463 6.976689 -1.956794

H 1.842309 6.279611 -3.206646

H -2.842651 5.783588 3.038999

H -4.620201 5.894011 1.760491

H -6.773786 -1.713202 1.463109

H -7.000392 -2.438185 -0.600292

H 1.729617 -0.881208 -6.414875

H 3.768385 -0.109947 -6.339992

**1*_m_***

H -0.815036 0.577161 1.708061

H -1.128627 1.194614 -0.842701

Ag -2.459282 -1.571948 -0.569679

Ag -1.340336 0.379605 -2.604442

Ag 0.205827 -1.962834 -1.733823

Ag -0.145648 -2.585315 1.092625

Ag -2.167260 -0.812429 2.261962

Ag -2.880721 1.315975 0.248075

Ag 0.052357 2.855249 -0.980288

Ag 1.625049 0.582146 -1.940414

Ag 2.316117 -1.157891 0.326159

Ag 0.823870 -0.273864 2.765493

Ag -0.573954 2.531336 1.946150

Ag 2.080797 1.711373 0.880752

Ag 2.543135 -3.049262 2.698739

Ag 1.921151 -4.217474 -0.565099

Ag 3.294856 -1.988129 -2.417562

Ag 1.329047 4.811558 0.988107

Ag 3.083654 3.301658 -1.565516

Ag -5.036538 -0.811237 0.995379

Ag -4.487482 0.150153 -2.264381

Rh -0.259333 0.181050 0.167232

S -3.013957 -2.349085 4.161112

S -4.825267 0.460631 3.217882

S -2.185452 4.362165 2.869942

S 1.216769 4.423807 3.520223

S 1.243044 -0.504812 5.266502

S 4.094883 -1.120541 3.338614

S 0.360162 -4.131733 3.506626

S -0.286827 -5.319995 0.291527

S -4.588983 -3.303841 0.483573

S -4.003865 -2.359870 -2.815222

S -6.612990 0.390816 -0.731007

S -4.575091 3.189689 0.005305

S -0.518133 5.523657 -0.723208

S 1.312554 4.121078 -3.327779

S 4.396035 2.303016 1.873328

S 3.734730 5.281500 0.065925

S 3.943999 -4.660235 1.086254

S 4.905795 -1.542593 -0.383182

S 2.983387 -4.501053 -2.975405

S -0.421663 -3.753475 -3.490524

S -3.605928 2.143052 -3.622245

S -1.604759 -0.284134 -5.088482

S 5.062662 1.742598 -2.093057

S 2.889965 -0.086302 -4.169417

P -4.532418 -1.085725 4.507164

P -0.657682 5.103569 3.930529

P 3.235843 -0.411205 5.038052

P 0.123772 -5.695345 2.239666

P -5.130739 -3.371758 -1.465875

P -6.351900 2.264201 0.050139

P 0.587139 5.769550 -2.410717

P 4.693459 4.258609 1.561130

P 5.349662 -3.362786 0.402005

P 1.344546 -4.349383 -4.202480

P -2.887145 1.245307 -5.299453

P 4.560694 1.068848 -3.908270

H 1.753142 -3.573653 -5.320308

H 1.197642 -5.627028 -4.805226

H -3.963277 0.853083 -6.143785

H -2.324605 2.286523 -6.088133

H -7.296327 3.109113 -0.589757

H -0.208880 6.478625 -3.348399

H 1.627067 6.708745 -2.173757

H -6.875670 2.213104 1.369193

H -6.501335 -3.024720 -1.613520

H -5.157056 -4.734745 -1.862602

H -0.924325 -6.504394 2.750980

H 1.226300 -6.586499 2.344129

H -4.420974 -0.493849 5.795202

H -5.745586 -1.810780 4.672670

H -0.842864 4.893821 5.324687

H -0.673660 6.525993 3.891037

H 3.707759 0.921662 5.176438

H 3.844799 -1.021568 6.169507

H 4.469064 5.011991 2.744736

H 6.082082 4.482006 1.361929

H 6.271129 -3.137571 1.456411

H 6.153165 -4.071950 -0.528686

H 5.663479 0.356900 -4.450290

H 4.428894 2.121018 -4.853284

**2*_m_***

H 0.459843 0.775003 1.699146

H -1.470081 -0.171173 1.444956

Ag -1.620810 -2.304515 -0.717354

Ag -1.502955 0.169681 -2.289284

Ag 0.898130 -1.453470 -1.926758

Ag 1.023931 -2.660569 0.719146

Ag -1.451830 -1.996949 2.198762

Ag -3.085737 0.190003 0.209617

Ag -1.318924 2.508626 -0.555098

Ag 1.105557 1.438940 -1.811904

Ag 2.609024 -0.179352 0.212401

Ag 1.145648 -0.684469 2.866993

Ag -1.416507 1.757880 2.333626

Ag 1.293305 2.455821 0.959748

Ag 3.752984 -2.254068 2.241687

Ag 3.470813 -2.915075 -1.157452

Ag 3.711200 -0.082426 -2.617867

Ag -0.834261 4.728868 1.516843

Ag 1.056343 4.504774 -1.307661

Ag -4.232035 -2.789058 0.851424

Ag -4.279601 -1.221588 -2.296835

Rh -0.197114 -0.007160 0.385689

S -1.578242 -4.071968 3.713120

S -4.331255 -1.997458 3.280137

S -3.432410 2.553175 3.756495

S -0.332130 4.082827 3.954672

S 1.857180 -0.818801 5.270803

S 5.020440 -0.612489 3.748978

S 2.266814 -4.307933 2.776945

S 1.970095 -4.984957 -0.632975

S -2.819425 -4.731122 -0.140594

S -2.912193 -3.203987 -3.267654

S -6.198484 -2.074173 -0.712497

S -5.424470 1.149644 0.542160

S -2.974861 4.617661 0.054234

S -1.027093 4.563005 -2.853181

S 3.206958 3.916154 1.819082

S 0.983977 6.375574 0.575008

S 5.630217 -2.659034 0.345905

S 4.988103 0.764969 -0.483885

S 4.400596 -2.386828 -3.582488

S 0.948299 -2.988259 -3.998671

S -4.302699 1.215562 -3.099847

S -1.584045 0.020720 -4.907697

S 3.495993 3.982542 -1.950370

S 2.363667 1.529608 -4.177080

P -3.443065 -3.551333 4.245759

P -2.205661 3.814549 4.708977

P 3.617529 0.129838 4.998567

P 2.594978 -5.546692 1.212563

P -3.451373 -4.742284 -2.076392

P -6.693116 -0.384726 0.335128

P -2.368569 5.502971 -1.674077

P 2.449670 5.772665 1.868534

P 6.222053 -0.728760 0.144515

P 2.760488 -2.681227 -4.780826

P -3.257226 1.118532 -4.847523

P 3.311820 3.304485 -3.827619

H 2.752170 -1.614277 -5.718838

H 3.089920 -3.776336 -5.622764

H -4.128426 0.728166 -5.903516

H -3.009677 2.469444 -5.216075

H -7.896387 0.103723 -0.239533

H -3.533561 5.741947 -2.448273

H -1.947258 6.831783 -1.398037

H -7.160656 -0.840104 1.596637

H -4.856489 -4.953501 -2.115632

H -2.983537 -5.948441 -2.661148

H 1.977777 -6.789636 1.511216

H 3.962307 -5.932228 1.164294

H -3.504849 -3.230148 5.629189

H -4.292395 -4.692360 4.217874

H -2.015000 3.435930 6.066201

H -2.833747 5.078271 4.890147

H 3.386243 1.493853 4.670892

H 4.214895 0.270961 6.279973

H 1.985927 6.105248 3.169336

H 3.525611 6.686532 1.717677

H 6.760291 -0.302662 1.385208

H 7.366074 -0.723398 -0.695514

H 4.603508 3.245739 -4.415593

H 2.673231 4.245089 -4.679142

**3*_m_***

H -0.373026 0.632282 1.831291

H -0.466629 -1.613225 0.994455

Ag -1.695274 -2.550407 -0.312695

Ag -1.381969 -0.479271 -2.385326

Ag 1.012150 -1.915068 -1.508721

Ag 1.375564 -2.364153 1.412199

Ag -1.784804 -1.012019 2.491872

Ag -3.063011 0.059116 -0.002262

Ag -1.314249 2.170572 -1.098133

Ag 1.145090 0.971575 -2.036699

Ag 2.715484 -0.044280 0.231186

Ag 1.317521 0.260086 2.817259

Ag -1.788169 1.978502 1.834490

Ag 1.086844 2.394275 0.656793

Ag 3.985396 -1.418352 2.772081

Ag 3.722827 -2.987016 -0.494225

Ag 3.826070 -0.548906 -2.528325

Ag -1.089079 4.749025 0.569893

Ag 1.088280 4.079372 -1.924596

Ag -4.362722 -2.546540 1.387121

Ag -4.131985 -1.920610 -2.244160

Rh -0.178969 -0.103105 0.335529

S -2.560412 -1.556845 4.786932

S -5.777584 -1.772700 3.374622

S -3.985291 3.026249 2.748325

S -0.914006 4.573769 3.134805

S 2.040402 0.717433 5.172766

S 5.304839 0.466822 3.893417

S 2.512538 -3.366846 3.717280

S 2.368933 -4.937929 0.616532

S -2.883713 -4.675958 1.059160

S -2.719204 -4.138587 -2.363782

S -6.134205 -2.342521 -0.635226

S -5.379766 1.095552 -0.358443

S -3.062646 4.284155 -1.051217

S -0.814318 3.776316 -3.679344

S 2.911256 4.051955 1.360012

S 0.811396 6.255143 -0.426219

S 5.805283 -2.304760 0.978815

S 5.086216 0.800439 -0.647951

S 4.606311 -2.971324 -2.994767

S 1.244162 -4.004488 -2.990590

S -3.911343 0.137406 -3.757897

S -1.090994 -1.561962 -4.813977

S 3.560367 3.564477 -2.451903

S 2.482130 0.792774 -4.322188

P -4.452498 -0.860936 4.589204

P -2.868310 4.412951 3.673777

P 3.828411 1.553638 4.739466

P 3.022925 -4.934065 2.532374

P -3.451858 -5.218000 -0.810994

P -6.657520 -0.431507 -0.121315

P -2.304323 4.854516 -2.852244

P 2.130540 5.880148 1.089946

P 6.359775 -0.473165 0.301928

P 2.943687 -3.657622 -3.982014

P -2.748088 -0.549925 -5.289038

P 3.406898 2.615916 -4.208049

H 2.739126 -2.799889 -5.095255

H 3.344278 -4.855139 -4.630865

H -3.549349 -1.301757 -6.193980

H -2.468455 0.587967 -6.094972

H -7.843653 -0.124821 -0.839438

H -3.388026 4.878374 -3.768074

H -1.960295 6.232437 -2.801010

H -7.127864 -0.505858 1.216934

H -4.868426 -5.302164 -0.886328

H -3.089560 -6.577844 -1.000146

H 2.556803 -6.119414 3.159519

H 4.428225 -5.139459 2.581990

H -4.413884 0.531629 4.300191

H -5.010059 -0.858141 5.895306

H -2.864578 4.228764 5.083280

H -3.505527 5.681817 3.588257

H 3.629638 2.743375 3.985623

H 4.330089 2.087831 5.956277

H 1.524446 6.367552 2.278680

H 3.205319 6.794286 0.932296

H 6.902379 0.247749 1.395972

H 7.493810 -0.656863 -0.531727

H 4.704729 2.483196 -4.769263

H 2.767656 3.413339 -5.194416

**4*_m_***

H -0.048713 -0.898078 1.732333

H -1.359216 0.779536 1.278521

Ag -2.147392 -2.022959 -0.407142

Ag -1.523422 0.152154 -2.329646

Ag 0.470015 -1.904553 -1.714542

Ag 0.433220 -2.734964 1.083000

Ag -1.994023 -1.037732 2.394665

Ag -3.033473 0.786109 0.093337

Ag -0.752269 2.558595 -0.780742

Ag 1.296579 0.881391 -2.009980

Ag 2.523983 -0.707294 0.154171

Ag 1.356231 0.092403 2.804931

Ag -0.621494 2.296065 2.285378

Ag 1.814162 2.105749 0.690542

Ag 3.331236 -2.454079 2.538677

Ag 2.701537 -3.758686 -0.713875

Ag 3.455572 -1.317086 -2.627664

Ag 0.272352 4.919627 0.891295

Ag 1.985410 3.869090 -1.841183

Ag -4.783246 -1.660478 1.155142

Ag -4.505894 -0.740233 -2.191913

Rh -0.206534 0.023730 0.356802

S -2.386216 -2.737471 4.345451

S -4.679346 -0.342544 3.357223

S -2.633624 3.437968 3.587542

S 0.601480 4.632664 3.426782

S 1.752709 -0.136660 5.337235

S 4.503751 -0.282929 3.208776

S 1.497767 -3.970425 3.515924

S 0.899786 -5.345328 0.373886

S -3.843502 -4.023760 0.635559

S -3.607023 -3.124720 -2.719142

S -6.572445 -0.836800 -0.564103

S -5.084719 2.305556 0.150294

S -1.875002 4.982087 -0.586490

S -0.020517 4.188632 -3.459208

S 3.999305 3.315884 1.382288

S 2.352332 5.977014 -0.287311

S 4.882883 -3.758262 0.805479

S 5.076180 -0.520507 -0.689640

S 3.640135 -3.848347 -3.187249

S 0.123987 -3.814696 -3.422504

S -4.020016 1.443303 -3.454685

S -1.593615 -0.586277 -4.873917

S 4.236483 2.717399 -2.371794

S 2.511553 0.397530 -4.351539

P -4.107401 -1.777934 4.689325

P -1.290094 4.784717 4.186396

P 3.724343 0.079350 5.052838

P 1.560074 -5.571681 2.280994

P -4.462576 -4.263528 -1.285786

P -6.672460 1.087326 0.133104

P -1.129871 5.502430 -2.413569

P 3.736889 5.270593 1.049655

P 5.945318 -2.198990 0.052299

P 1.912719 -4.016062 -4.284512

P -3.036068 0.779159 -5.112332

P 3.877733 1.907100 -4.169085

H 2.061711 -3.150428 -5.400832

H 1.981993 -5.285227 -4.918079

H -3.977797 0.330076 -6.081068

H -2.553976 1.949125 -5.761298

H -7.715663 1.719579 -0.594045

H -2.242492 5.872572 -3.212965

H -0.458541 6.748534 -2.287093

H -7.260794 0.994718 1.422660

H -5.880085 -4.176844 -1.346673

H -4.264380 -5.627860 -1.625486

H 0.797684 -6.611937 2.873516

H 2.855593 -6.156230 2.278066

H -4.102762 -1.137006 5.958526

H -5.172684 -2.701352 4.882442

H -1.123755 4.782424 5.598991

H -1.765421 6.112639 3.997208

H 4.158124 1.389269 5.392328

H 4.426240 -0.680458 6.029751

H 3.488923 5.969543 2.261597

H 4.978432 5.841911 0.662217

H 6.858916 -1.780679 1.053157

H 6.822993 -2.728661 -0.929457

H 5.108981 1.465070 -4.722644

H 3.487460 2.879122 -5.128582

**TS*_m_*1**

H -0.173148 0.918516 1.653471

H -1.317205 0.856436 -0.904675

Ag -2.031191 -2.094452 -0.433821

Ag -1.314117 -0.057175 -2.642229

Ag 0.672531 -1.935832 -1.670412

Ag 0.390510 -2.499360 1.213651

Ag -1.889459 -0.847937 2.245715

Ag -3.180720 0.605624 0.033830

Ag -0.569035 2.747911 -1.136866

Ag 1.531493 0.880790 -1.983913

Ag 2.521987 -0.642380 0.348446

Ag 0.978068 -0.124853 2.835877

Ag -1.477029 2.283876 1.769871

Ag 1.665705 2.163885 0.796433

Ag 3.095146 -2.462582 2.865722

Ag 2.862244 -3.701087 -0.433538

Ag 3.812263 -1.215352 -2.306109

Ag 0.043311 4.934035 0.846779

Ag 2.267659 3.891159 -1.626327

Ag -4.686107 -1.864171 1.169362

Ag -4.328961 -1.105627 -2.392558

Rh -0.235636 0.138805 0.138026

S -2.741674 -1.321814 4.563901

S -5.924573 -0.883426 3.168589

S -3.391825 3.759892 2.607751

S -0.114420 4.609599 3.350710

S 1.558122 0.072553 5.268084

S 4.706988 -1.088539 4.238720

S 1.018338 -3.929785 3.496340

S 0.865549 -5.283156 0.287586

S -3.564175 -4.193559 0.747963

S -3.209668 -3.442692 -2.624563

S -6.411848 -1.300943 -0.877340

S -5.220361 2.017073 -0.410676

S -1.783686 5.143513 -0.991207

S 0.425517 4.257646 -3.525051

S 3.800679 3.252215 1.762958

S 2.328343 5.947583 0.024431

S 4.907831 -3.635816 1.194925

S 5.109000 -0.362773 -0.214191

S 4.047564 -3.754391 -2.797520

S 0.585192 -3.875152 -3.376674

S -3.813429 0.951284 -3.857304

S -1.197428 -1.009363 -5.013105

S 4.580942 2.879621 -2.071136

S 3.014170 0.517030 -4.145708

P -4.436650 -0.241118 4.371161

P -2.104321 4.866328 3.684008

P 3.543566 0.356584 5.046480

P 1.172521 -5.537625 2.254099

P -4.105847 -4.535586 -1.170082

P -6.666439 0.628098 -0.233464

P -0.709673 5.666874 -2.645520

P 3.454769 5.212401 1.567999

P 5.942551 -1.962372 0.736555

P 2.453372 -3.981156 -4.070567

P -2.773692 0.176620 -5.433006

P 4.301889 2.082180 -3.887352

H 2.670708 -3.095078 -5.159317

H 2.639354 -5.237168 -4.706560

H -3.648234 -0.490542 -6.335455

H -2.392615 1.291126 -6.228457

H -7.813823 1.128538 -0.903083

H -1.637294 6.169611 -3.595487

H 0.048762 6.835150 -2.363835

H -7.103900 0.537923 1.114377

H -5.519468 -4.464211 -1.302234

H -3.878445 -5.907690 -1.455964

H 0.254800 -6.507936 2.736080

H 2.406613 -6.197087 2.505269

H -4.122120 1.112673 4.066011

H -4.997234 -0.097872 5.668332

H -2.296372 4.676386 5.079575

H -2.447494 6.241677 3.570629

H 3.791713 1.583153 4.370422

H 4.065299 0.651707 6.334069

H 2.901002 5.771077 2.751118

H 4.704119 5.884286 1.508027

H 6.515931 -1.452028 1.928931

H 7.089537 -2.353146 -0.002885

H 5.562602 1.698454 -4.417567

H 3.895465 3.051718 -4.842223

**TS*_m_*2**

H -0.298000 0.578000 1.861000

H -1.729000 -0.720000 0.692000

Ag -1.684000 -2.411000 -0.567000

Ag -1.462000 0.014000 -2.390000

Ag 0.853000 -1.643000 -1.840000

Ag 0.984000 -2.548000 0.934000

Ag -1.402000 -1.654000 2.413000

Ag -3.192000 0.468000 -0.079000

Ag -1.210000 2.547000 -0.828000

Ag 1.155000 1.267000 -1.916000

Ag 2.595000 -0.216000 0.165000

Ag 1.248000 -0.241000 2.793000

Ag -1.689000 1.944000 2.038000

Ag 1.216000 2.292000 0.949000

Ag 3.751000 -2.106000 2.399000

Ag 3.392000 -3.108000 -1.005000

Ag 3.716000 -0.369000 -2.650000

Ag -0.736000 4.801000 1.197000

Ag 1.350000 4.298000 -1.405000

Ag -4.324000 -2.548000 1.232000

Ag -4.244000 -1.564000 -2.203000

Rh -0.289000 0.012000 0.285000

S -1.557000 -3.358000 4.306000

S -4.281000 -1.359000 3.476000

S -3.669000 2.944000 3.321000

S -0.522000 4.361000 3.712000

S 2.062000 -0.102000 5.162000

S 5.250000 -0.540000 3.761000

S 2.130000 -4.033000 3.048000

S 1.829000 -5.060000 -0.271000

S -2.970000 -4.669000 0.538000

S -2.881000 -3.682000 -2.794000

S -6.208000 -2.147000 -0.539000

S -5.528000 1.098000 0.718000

S -2.773000 4.818000 -0.423000

S -0.613000 4.431000 -3.127000

S 3.208000 3.674000 1.784000

S 1.283000 6.270000 0.360000

S 5.542000 -2.819000 0.487000

S 5.029000 0.558000 -0.578000

S 4.311000 -2.748000 -3.464000

S 0.854000 -3.334000 -3.795000

S -4.533000 0.784000 -3.213000

S -1.760000 -0.440000 -4.930000

S 3.767000 3.693000 -2.098000

S 2.439000 1.260000 -4.249000

P -3.406000 -2.696000 4.732000

P -2.473000 4.255000 4.252000

P 3.912000 0.597000 4.762000

P 2.454000 -5.432000 1.618000

P -3.549000 -4.981000 -1.379000

P -6.754000 -0.201000 -0.188000

P -1.923000 5.575000 -2.103000

P 2.572000 5.578000 1.793000

P 6.215000 -0.940000 0.126000

P 2.651000 -3.081000 -4.628000

P -3.495000 0.567000 -4.950000

P 3.480000 2.997000 -3.948000

H 2.639000 -2.062000 -5.613000

H 2.954000 -4.220000 -5.417000

H -4.350000 0.012000 -5.950000

H -3.333000 1.885000 -5.465000

H -7.203000 0.292000 -1.442000

H -2.981000 5.928000 -2.986000

H -1.363000 6.852000 -1.824000

H -7.963000 -0.245000 0.548000

H -4.961000 -5.106000 -1.459000

H -3.133000 -6.284000 -1.756000

H 1.828000 -6.630000 2.051000

H 3.820000 -5.826000 1.619000

H -3.448000 -2.098000 6.019000

H -4.270000 -3.808000 4.945000

H -2.476000 4.027000 5.656000

H -3.056000 5.554000 4.214000

H 3.819000 1.871000 4.136000

H 4.494000 0.948000 6.008000

H 2.005000 5.933000 3.047000

H 3.712000 6.416000 1.755000

H 6.803000 -0.453000 1.319000

H 7.327000 -1.051000 -0.743000

H 4.742000 2.861000 -4.585000

H 2.856000 3.951000 -4.792000

**TS*_m_*3**

H -1.439000 0.823000 1.113000

H -0.523000 -1.209000 1.393000

Ag -2.006000 -2.192000 -0.213000

Ag -1.546000 -0.176000 -2.400000

Ag 0.600000 -1.990000 -1.618000

Ag 0.847000 -2.620000 1.226000

Ag -2.013000 -0.588000 2.588000

Ag -3.127000 0.574000 -0.104000

Ag -0.952000 2.429000 -0.961000

Ag 1.191000 0.841000 -2.090000

Ag 2.578000 -0.500000 0.158000

Ag 1.143000 0.102000 2.709000

Ag -0.806000 2.301000 2.228000

Ag 1.603000 2.202000 0.595000

Ag 3.534000 -2.005000 2.709000

Ag 3.107000 -3.552000 -0.645000

Ag 3.588000 -1.118000 -2.619000

Ag -0.130000 4.894000 0.669000

Ag 1.681000 3.885000 -1.989000

Ag -4.682000 -1.808000 1.369000

Ag -4.400000 -1.412000 -2.109000

Rh -0.250000 -0.006000 0.287000

S -2.056000 -2.343000 4.583000

S -4.657000 -0.356000 3.484000

S -2.918000 3.324000 3.495000

S 0.205000 4.766000 3.220000

S 1.921000 0.326000 5.092000

S 5.118000 -0.316000 3.793000

S 1.812000 -3.749000 3.605000

S 1.461000 -5.251000 0.490000

S -3.484000 -4.109000 1.068000

S -3.371000 -3.769000 -2.398000

S -6.494000 -1.417000 -0.487000

S -5.308000 1.784000 0.448000

S -2.270000 4.771000 -0.803000

S -0.336000 4.048000 -3.632000

S 3.752000 3.530000 1.163000

S 1.912000 6.060000 -0.481000

S 5.206000 -3.180000 0.910000

S 5.043000 -0.011000 -0.733000

S 3.970000 -3.630000 -3.134000

S 0.465000 -4.000000 -3.221000

S -4.344000 0.747000 -3.518000

S -1.673000 -0.991000 -4.897000

S 4.006000 2.965000 -2.639000

S 2.415000 0.383000 -4.405000

P -3.879000 -1.598000 4.915000

P -1.696000 4.825000 3.972000

P 3.799000 0.939000 4.670000

P 2.124000 -5.350000 2.405000

P -4.116000 -4.705000 -0.775000

P -6.700000 0.619000 -0.399000

P -1.538000 5.311000 -2.629000

P 3.289000 5.462000 0.911000

P 6.074000 -1.474000 0.237000

P 2.227000 -3.981000 -4.161000

P -3.317000 0.117000 -5.158000

P 3.615000 2.042000 -4.370000

H 2.226000 -3.092000 -5.269000

H 2.409000 -5.228000 -4.815000

H -4.207000 -0.544000 -6.052000

H -3.045000 1.295000 -5.907000

H -6.990000 1.033000 -1.726000

H -2.662000 5.597000 -3.448000

H -0.949000 6.600000 -2.519000

H -7.937000 0.876000 0.248000

H -5.536000 -4.725000 -0.801000

H -3.827000 -6.091000 -0.886000

H 1.521000 -6.482000 3.013000

H 3.493000 -5.730000 2.430000

H -3.940000 -0.859000 6.128000

H -4.814000 -2.630000 5.206000

H -1.534000 4.957000 5.379000

H -2.277000 6.089000 3.670000

H 3.760000 2.159000 3.939000

H 4.374000 1.380000 5.892000

H 2.901000 6.069000 2.136000

H 4.482000 6.174000 0.621000

H 6.713000 -0.849000 1.338000

H 7.174000 -1.849000 -0.578000

H 4.843000 1.703000 -4.997000

H 3.054000 2.920000 -5.336000

**TS*_m_*4**

H -1.183996 1.310420 0.846215

H -0.251360 -0.473638 1.886072

Ag -2.252748 -1.878278 -0.427942

Ag -1.511457 0.309766 -2.288951

Ag 0.341673 -1.878839 -1.748253

Ag 0.289951 -2.599953 1.115283

Ag -2.093652 -0.997913 2.348124

Ag -3.031169 0.919976 0.152116

Ag -0.571635 2.705673 -0.783102

Ag 1.342348 0.872473 -2.038757

Ag 2.486867 -0.855792 0.053331

Ag 1.518835 -0.144822 2.743109

Ag -0.366649 2.157477 2.448946

Ag 1.972674 1.971887 0.692941

Ag 3.214470 -2.767958 2.347339

Ag 2.411197 -3.875144 -0.804733

Ag 3.343485 -1.447117 -2.749234

Ag 0.603434 4.816974 1.147989

Ag 2.189905 3.808139 -1.773497

Ag -4.924345 -1.456292 1.104820

Ag -4.563808 -0.396494 -2.171169

Rh -0.194486 0.102907 0.324132

S -2.345889 -2.992540 4.112194

S -4.638562 -0.489118 3.465056

S -2.253773 3.263922 3.927248

S 0.997722 4.408873 3.650173

S 1.765410 -0.499009 5.319446

S 4.472162 -0.679112 3.160140

S 1.232814 -4.094075 3.318753

S 0.484260 -5.353063 0.154329

S -4.055423 -3.801315 0.337940

S -3.821037 -2.724856 -2.973093

S -6.659293 -0.429534 -0.562299

S -5.018175 2.318634 0.956429

S -1.563183 5.179444 -0.262353

S 0.105275 4.411158 -3.231727

S 4.244233 3.000794 1.388662

S 2.683448 5.807303 -0.107829

S 4.678322 -4.080344 0.576707

S 5.033258 -0.802394 -0.810776

S 3.309932 -3.979649 -3.301992

S -0.189187 -3.610593 -3.591796

S -4.096764 2.000605 -3.010669

S -1.850815 0.113906 -4.875190

S 4.352472 2.574285 -2.447410

S 2.476947 0.359019 -4.415215

P -4.076000 -2.110691 4.580040

P -0.859851 4.578815 4.485278

P 3.744589 -0.434316 5.050133

P 1.069718 -5.680421 2.060483

P -4.695360 -3.906529 -1.591962

P -6.571550 1.551676 -0.046280

P -0.783769 5.778157 -2.043909

P 4.096715 4.976704 1.121679

P 5.813126 -2.551614 -0.134900

P 1.585287 -4.009371 -4.415559

P -3.162047 1.621458 -4.780076

P 3.904395 1.811195 -4.245501

H 1.832580 -3.193002 -5.551759

H 1.541152 -5.294889 -5.018112

H -4.135432 1.477446 -5.808623

H -2.568731 2.854885 -5.164528

H -6.822317 2.269380 -1.246116

H -1.837064 6.381329 -2.779799

H 0.072773 6.892225 -1.832726

H -7.752165 1.829912 0.691607

H -6.110015 -3.771961 -1.624692

H -4.545780 -5.256302 -2.005931

H 0.149889 -6.587850 2.647359

H 2.264321 -6.450139 2.092857

H -4.093497 -1.666499 5.931236

H -5.146587 -3.047870 4.617727

H -0.640265 4.551626 5.890120

H -1.321100 5.916214 4.333461

H 4.301367 0.791663 5.505114

H 4.392201 -1.346224 5.929897

H 3.966947 5.654365 2.363695

H 5.347804 5.479598 0.674385

H 6.759291 -2.217283 0.867329

H 6.650435 -3.093335 -1.144830

H 5.101671 1.333790 -4.842277

H 3.523770 2.814984 -5.175570
